# Organophosphorus flame retardants are developmental neurotoxicants in a rat primary brainsphere in vitro model

**DOI:** 10.1007/s00204-020-02903-2

**Published:** 2020-10-19

**Authors:** Helena T. Hogberg, Rita de Cássia da Silveira E Sá, Andre Kleensang, Mounir Bouhifd, Ozge Cemiloglu Ulker, Lena Smirnova, Mamta Behl, Alexandra Maertens, Liang Zhao, Thomas Hartung

**Affiliations:** 1 Center for Alternatives To Animal Testing (CAAT), Johns Hopkins Bloomberg School of Public Health, Baltimore, MD USA; 2 Department of Physiology and Pathology, Federal University of Paraíba, João Pessoa, Brazil; 3 Department of Toxicology, Faculty of Pharmacy, Ankara University, Ankara, Turkey; 4 National Toxicology Program, National Institute of Environmental Health Sciences, Research Triangle Park, Durham, NC USA; 5 Bloomberg~Kimmel Institute for Cancer Immunotherapy, Johns Hopkins University School of Medicine, Baltimore, MD USA; 6 CAAT-Europe, University of Konstanz, Konstanz, Germany

**Keywords:** Developmental neurotoxicity, Flame retardants, 3D in vitro model, New approach methodologies, Metabolomics, Transcriptomics

## Abstract

Due to regulatory bans and voluntary substitutions, halogenated polybrominated diphenyl ether (PBDE) flame retardants (FR) are increasingly substituted by mainly organophosphorus FR (OPFR). Leveraging a 3D rat primary neural organotypic in vitro model (rat brainsphere), we compare developmental neurotoxic effects of BDE-47—the most abundant PBDE congener—with four OPFR (isopropylated phenyl phosphate—IPP, triphenyl phosphate—TPHP, isodecyl diphenyl phosphate—IDDP, and tricresyl phosphate (also known as trimethyl phenyl phosphate)—TMPP). Employing mass spectroscopy-based metabolomics and transcriptomics, we observe at similar human-relevant non-cytotoxic concentrations (0.1–5 µM) stronger developmental neurotoxic effects by OPFR. This includes toxicity to neurons in the low µM range; all FR decrease the neurotransmitters glutamate and GABA (except BDE-47 and TPHP). Furthermore, *n*-acetyl aspartate (NAA), considered a neurologic diagnostic molecule, was decreased by all OPFR. At similar concentrations, the FR currently in use decreased plasma membrane dopamine active transporter expression, while BDE-47 did not. Several findings suggest astrogliosis induced by the OPFR, but not BDE-47. At the 5 µM concentrations, the OPFR more than BDE-47 interfered with myelination. An increase of cytokine gene and receptor expressions suggests that exposure to OPFR may induce an inflammatory response. Pathway/category overrepresentation shows disruption in 1) transmission of action potentials, cell–cell signaling, synaptic transmission, receptor signaling, (2) immune response, inflammation, defense response, (3) cell cycle and (4) lipids metabolism and transportation. Taken together, this appears to be a case of regretful substitution with substances not less developmentally neurotoxic in a primary rat 3D model.

**Electronic supplementary material:**

The online version of this article (10.1007/s00204-020-02903-2) contains supplementary material, which is available to authorized users.

## Introduction

Flame retardants (FR) are a group of compounds, which are added to consumer products, including upholstered furniture, electrical devices, baby products, textiles and plastics, to restrain or delay flame propagation to prevent fire spreading (Dishaw et al. 2014b; EPA US 2005; Jarema et al. 2015). The global FR consumption has surpassed 2 million tons and is yet expected to increase due to international flammability standards (Ceresana 2018). However, FR exhibit characteristics similar to environmental toxicants, such as heavy metals, air pollutants and pesticides that are well recognized as hazardous to human health and inducers of neurodevelopmental damage. Prior to 2005, halogenated polybrominated diphenyl ethers (PBDEs) were the primary FR used in the USA. However, the halogenated FR have been linked to the development of cancer, endocrine disruption, immunotoxicity, reproductive toxicity, and fetal and child development perturbation (Birnbaum and Staskal 2004; Costa and Giordano 2007; Roze et al. 2009; Shaw et al. 2010). In particular, 2,2′,4,4′-tetrabromodiphenyl ether (BDE-47)—the most abundant PBDE congener in the environment and human serum (Birnbaum and Staskal 2004; EPA US 2008)—has been shown to affect the adult and developing nervous system (Dingemans et al. 2007; Eriksson et al. 2002).

Due to these human health concerns, PBDEs have been banned in Europe and phased out in the USA (Birnbaum and Staskal 2004; Feo et al. 2013), and mainly been replaced by organophosphorus FR (OPFR) (Blum et al. 2019; Dishaw et al. 2014a, 2014b; Stapleton et al. 2014). Following the phase out of PBDEs, there is increasing evidence showing higher exposure to OPFRs compared to PBDEs from hand wipes in toddlers, and house dust suggesting that the magnitude of exposure via hand-to-mouth and dermal transfer pathways is potentially greater for OPFRs than for PBDEs (Blum et al. 2019).

The use of OPFRs is further anticipated to increase following the recent proposal by the European Commission to prohibit the class of organohalogen chemicals in electronic display enclosures and stands effective since April 2021 (Commission 2018). Hence, it is likely that the manufacturers will explore the potential for use of OPFRs in televisions and other electronics as alternative methods to meet flammability codes.

Although there has been an increase in the use of OPFRs, there is still relatively limited information on their potential health effects. This is of concern, as OPFRs bear some structural similarities to organophosphate pesticides that are well known to induce (developmental) neurotoxicity (Burke et al. 2017; Grandjean and Landrigan 2014; Mie et al. 2018). Especially, the use of FR in baby products and the exposure to children are of concern as the developing brain is much more vulnerable to environmental perturbation than the brain of adults (O'Rahilly and Muller 2008; Smirnova et al. 2014).

The exposure to industrial chemicals, including FR, and drugs during early development has been associated with the occurrence of neurodevelopmental disorders in children such as autism, mental retardation, dyslexia, epilepsy or mental deficit (Grandjean and Landrigan 2006, 2014; Rice and Barone 2000; Zhong et al. 2020).

Current DNT testing guidelines for chemicals and pesticides (EPA U 1998; OECD 2007, 2011) are based on traditional in vivo animal studies. These guidelines require elaborated, lengthy and costly study protocols that are incompatible with the assessment of large numbers of chemicals in addition to elicit uncertain predictivity for human risks (Smirnova et al. 2014). Hence, the screening of chemicals requires the employment of more reliable, cheap and fast tools capable of determining DNT potential to ensure the safety of children’s health (Bal-Price et al. 2015a, 2010, 2018; Bjorling-Poulsen et al. 2008; Fritsche et al. 2017).

The use of in vitro, *in silico* and non-mammalian species-based methodologies and models has been proposed to enhance the DNT assessment in terms of cost, time and mechanistic understanding (Bal-Price et al. 2015a, 2018; Coecke et al. 2007; Smirnova et al. 2014). Several promising in vitro cell models have been developed; however, most of them still have difficulties in simulating the complex structure of the developing brain. Three-dimensional (3D) primary organotypic cell cultures have the advantage of reproducing the complex multicellular environment that closely resembles in vivo conditions (Alepee et al. 2014; Pamies et al. 2017; Sundstrom et al. 2005). The 3D rat primary neural organotypic in vitro model, used in this study, is a model able to recreate the CNS in vivo structural characteristics and biochemical signaling (Forsby et al. 2009; Honegger and Monnet-Tschudi 2001; van Vliet et al. 2007). It consists of most of the relevant cell types in the brain such as neurons, astrocytes, oligodendrocytes and microglia (Honegger et al. 1979). The model has been extensively characterized by immunohistochemistry, electrophysiology, pharmacological behavior and expression of neurodevelopment marker genes (van Vliet et al. 2007). The model is considered mature after 21–28 days in vitro (*DIV*), as electrical activity, synaptogenesis and myelination are robust at this time. The DNT consensus process identified the rat aggregating cell model among the most representative models for DNT studies (Bal-Price et al. 2010).

Taking into consideration the importance of identifying and characterizing potential DNT chemicals by applying suitable testing methods, this study aimed to investigate the DNT potential of four OPFR (isopropylated phenyl phosphate—IPP, triphenyl phosphate—TPHP, isodecyl diphenyl phosphate—IDDP, and tricresyl phosphate (also known as trimethyl phenyl phosphate)—TMPP) relative to one replaced PBDE (BDE-47) using metabolomics and transcriptomics approaches. The exposure to the FR induced significant alterations in gene expression and metabolite levels, with stronger effect after exposure to OPFRs. The major effects were observed for genes and metabolites associated with the neurotransmitter glutamate and its receptors, followed by general neuronal markers and reactivated glial cells. Many of the alterations could be linked to existing DNT adverse outcome pathways (AOPs) (Sachana et al. 2018; Spinu et al. 2019; Wang et al. 2018), which increase the concern of OPFRs usage as replacements of PBDE and imply the need for additional assessment of these compounds.

## Materials and methods

### Animals

Pregnant female Sprague–Dawley rats were used as the source of embryonic brain tissue for dissociation and re-aggregation in vitro. The animals were kept in the Johns Hopkins Bloomberg School of Public Health Animals Resources Facility for 48 h. They were housed individually under standard laboratory conditions with controlled temperature of 22 °C and a 12-h light/dark photoperiod. Food and water were supplied ad libitum. Housing and experimental protocols were approved by the Institutional Animal Care and Use Committee (IACUC) (Protocol RA15H122).

Sixteen-day pregnant females were anesthetized by inhalation of tribromoethanol (Sigma-Aldrich) following immediate decapitation to minimize any pain or distress. Aseptic conditions were maintained throughout the procedure to prevent contamination of tissue cell cultures. The rats were sterilized with 70% ethanol and an incision was made through the skin over the midline of the abdomen for the removal of the uterus containing the fetuses. The fetuses were then excised from the uterus and the whole brain was dissected out for the preparation of re-aggregating brain cell cultures.

### 3D rat primary neural organotypic in vitro model

3D rat primary neural cell cultures were prepared from 16-day-old fetal rat brains as described previously (van Vliet et al. 2008). Briefly, the brain tissue was mechanically dissociated in Puck’s solution [NaCl (9 g/l), KCl (0.4 g/l), Na_2_HPO_4_ (75 mg/l), KH_2_PO_4_, (30 mg/l), phenol red (5 mg/l), d-glucose monohydrate (1.1 g/l) and d-sucrose (20 g/l) in water, adjusted pH 7.4 with 0.2 N NaOH] (all from Sigma-Aldrich) following re-suspension of the cells at a density of 7.5 × 10^6^ cell/ml in a modified serum-free media: DMEM with high glucose (25 mM, Thermo Fisher Scientific) supplemented with insulin (0.8 µM), triiodothyronine (30 nM), hydrocortisone solution (20 nM), apo-transferrin (1 µg/ml), biotin (4 µM), vitamin B_12_ (1 µM), linoleic acid (10 µM), lipoic acid (1 µM), _L_-carnitine (10 µM) and trace elements [Na_2_SiO_3_ (2.5 mM), Na_2_SeO_3_ (1.5 mM), CdSO_4_ (0.5 mM), CuSO_4_ (1 mM), MnCl_2_ (0.5 mM), (NH_4_)MO_3_ (0.005 mM), NiSO_4_ (0.025 mM), SnCl_2_ (0.025 mM), ZnSO_4_ (0.5 mM)] (all from Sigma-Aldrich). Cells were re-aggregated and kept in glass flasks (15 ml initial, followed by 20 ml from *DIV*4) under gyratory shaking using an orbital shaker (Kühner shaker ES-X, 50 mm orbit diameter, Adolf Kühner AG, Birsfelden, CH). The initial speed was set to 68 rpm and was increased over 7 days to a constant speed of 80 rpm, at 37 °C in an atmosphere of 10% of CO_2_. Half of the culture media was replenished every 3 days. After 3 days in vitro (*DIV*3), 3D aggregates were formed, which were size-wise and morphologically robust (increasing in size from ~ 350 µm at *DIV*7 to ~ 400 µm at *DIV*21) (Trapp et al. 1979; van Vliet et al. 2007).

### Chemical treatments

Five FR (Table 1) and corresponding vehicle control (DMSO) were studied. FR were supplied by the Division of National Toxicology Program, U.S. National Institute of Environmental Health Sciences (NTP, NIEHS, Research Triangle Park, NC). Stock solutions were prepared in DMSO (Sigma-Aldrich). The final concentration of DMSO did not exceed 0.1% (v/v), which is non-toxic in the rat brainsphere model.

**Table 1:** Flame retardants (FR) evaluated in a 3D rat primary neural organotypic in vitro model

Flame Retardant	CAS	Supplier	CoA Purity	Conc. stock	Conc. well
2,2′4,4′Tetrabromodiphenyl ether (BDE-47)	5436-43-1	Cerilliant Corp	98%	20 mM	0.1–20 µM
Triphenyl phosphate (TPHP)	115-86-6	Acros Organics	99%	100 mM	0.1–20 µM
Isopropylated phenol phosphate (IPP)	68937-41-7	Chemtura	NA	100 mM	0.1–10 µM
Isodecyl diphenyl phosphate (IDDP)	29-761–21-5	Ferro Corp	NA	100 mM	0.1–20 µM
Tricresyl phosphate (TMPP)	1330–78-5	Acros Organics	99%	100 mM	0.1–20 µM
Dimethyl sulfoxide (DMSO)	67–68-5	Sigma-Aldrich	100%	NA	0.1% (v/v)

**Fig. 1:**
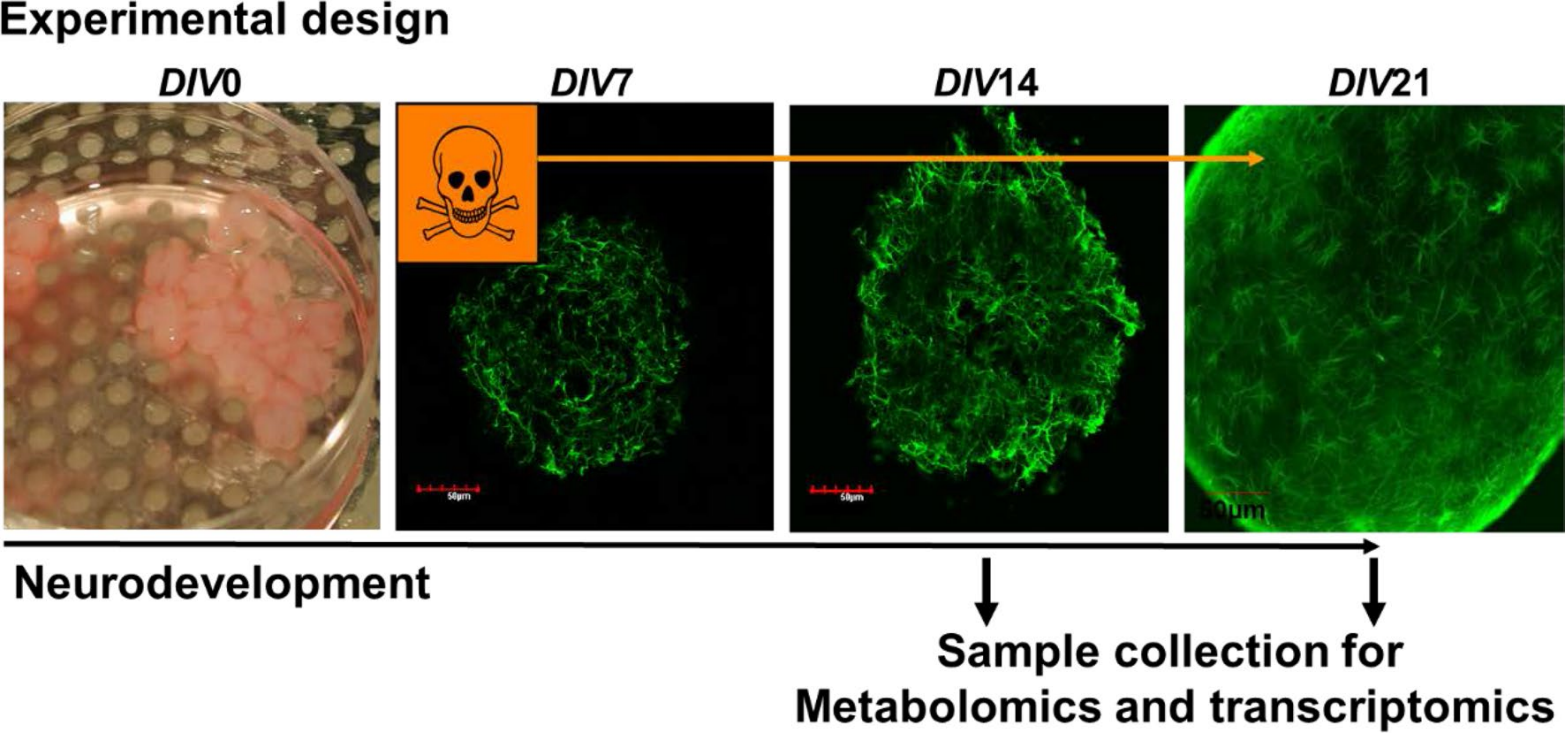
Schematic experimental design of rat brainsphere model exposed during development to FR at *DIV*7. Samples were collected at *DIV*14 and 21 for metabolomics and transcriptomics analysis

At *DIV*7, aggregating cell cultures were pooled and distributed over six-well plates, each containing approximately 100 aggregates in 2 ml of serum-free media. The cultures were then exposed to the chemicals up to *DIV*14 and *DIV*21, a period that covers crucial developmental processes and different stages of cell maturation (Fig. 1). The concentrations of FR were chosen based on preliminary range-finding experiments, where wide ranges of concentrations were tested using cell viability assay (resazurin). In final experiments, three non-cytotoxic concentrations at *DIV*14 (0.1, 1, and 5 µM) were selected for metabolomics and gene expression analysis. Importantly, using in vitro to in vivo extrapolation suggests that these are relevant human exposures (Blum et al. 2019). Half of the culture media were exchanged with new chemical treatment twice per week. One non-cytotoxic concentration at *DIV*21 (1 µM) of IPP was selected for transcriptomics analyses.

### Assessment of cell viability

Cell viability was determined after exposure to the selected chemicals (0.1–20 µM) at *DIV*14 and *DIV*21 using the resazurin reduction assay (O'Brien et al. 2000). The blue colored dye resazurin is reduced to fluorescent resorufin by redox reactions in viable cells with active metabolism. Resazurin (0.01 mg/ml, Sigma-Aldrich) in PBS was added directly to the six-well plates, without removing the medium at the end of the period of exposure to the tested compounds. The plates were incubated for 2 h at 37 °C, 10% CO_2_. After incubation, 100 µl of medium from each sample was transferred to a 96-well plate and the fluorescence of the resazurin metabolite, resorufin, was measured at 530 nm/590 nm (excitation/emission) in a multi-well fluorometric reader (Cytofluor Multi-well Plate Reader Series 4000, Perseptive Biosystems). The differentiation medium was incubated with resazurin in parallel as a blank control. Cell viability was calculated as % of fluorescence intensity relative to solvent-treated controls after subtracting average blanks. Data are presented as mean ± SD of at least three independent experiments performed in two to four replicates. Differences between treated and DMSO control groups were assessed by one-way ANOVA (GraphPad Prism 8.4.3), followed by Dunnett’s multiple comparison post hoc test including correction for multiple testing. Post hoc test was only performed vs. controls. Statistical significance is indicated as follows: *p < 0.05 (treated vs*.* control).

### RNA purification, reverse transcription, and quantitative real-time PCR (RT-qPCR)

Cell samples were lysed for mRNA expression analysis and total RNA extraction was carried out according to the manufacturer’s protocol of RNeasy Mini Kit (Qiagen). RNA integrity was assessed with the Nanodrop 2000 (Thermo Scientific) UV–Vis spectrophotometer at 260 nm. Reverse transcription was performed as follows: 500 ng RNA was incubated with 2.5 mM PCR Nucleotide Mix (Promega) and 12.5 µg/ml random primers (Promega) for 5 min at 65 °C using a Techne PCR system. Subsequently, 2 units/µl RNaseOut inhibitor (Thermo Fisher Scientific), 10 units/µl Moloney murine leukemia virus (M-MLV) reverse transcriptase (Promega) and the samples were incubated for 10 min at 25 °C for annealing, 60 min at 37 °C for cDNA synthesis and 15 min at 70 °C for inactivation of enzymes. RNase-free DNase set (Qiagen) was used to avoid contamination with DNA. cDNA was diluted 1:5 and quantitative RT-PCR was performed using the Fast Applied Biosystems 7500 System (Life Technologies). Genes were selected (Table S1) based on previous studies (Hogberg et al. 2009, 2010) and transcriptomics data for IPP. The expression was measured using TaqMan gene expression assay (Life Technologies) and FastStart Universal Probe Master mix (ROX) (Roche) according to the manufactures protocol. Relative RNA quantification was performed using the comparative CT method, normalizing the data to a standard calibrator (a mixture of samples from the different time points of the cell proliferation and differentiation), and to the 18S rRNA content (Schmittgen and Livak 2008). Data were calculated and presented as average log_2_-fold change in each independent experiment ± SD of at least three independent experiments performed in two to three replicates. Differences between treated and non-treated groups were assessed by two-way ANOVA (GraphPad Prism 8.4.3), followed by Bonferroni’s comparison post hoc test including correction for multiple testing. Post hoc test was only performed vs. controls. Statistical significance is indicated as follows: *p < 0.05.

### Transcriptomics sample preparation and analyses

Perturbations in transcriptome were analyzed by microarray after exposure to 1 µM of IPP or solvent control (DMSO) from *DIV*7 to *DIV*21. Transcriptomics was performed in triplicate from one experiment. 100 ng of total RNA from treated and control cells was converted into cDNA and then into labeled cRNA using Agilent LowInput QuickAmp Labeling Kit (Agilent). The resulting cRNA was labeled with Cy3. Labeled cRNAs were then purified, and RNA concentration and dye incorporation were measured using Nanodrop 2000 spectrophotometer (Thermo Scientific). Hybridization to SurePrint G3 Rat Gene Expression 8 × 60 k (Agilent, Product No. G4853A, Grid No. 028279) was conducted following the manufacturer’s protocol. Microarrays were scanned with an Agilent DNA microarray scanner. Feature Extraction (12.0.0.7 version, Agilent) was used to calculate the signal intensity and ratios. All arrays met each of the ten quality evaluation metrics parameters of Feature Extraction (“Good”).

After deleting non-detected probes and quantile-normalization, statistical and pathway overrepresentation analyses were performed with activated mean centering and scaling option (GeneSpring V13.1, Agilent).

### Metabolomics sample preparation and analyses

Cells were collected in 1.5 ml Eppendorf tubes and washed three times with ice-cold PBS. After removal of PBS, ice-cold high-purity methanol (MeOH) (Sigma-Aldrich) was added. Cells were stored at – 80 ℃ until use. For metabolite extraction, 75 µl of HPLC-grade water was added to the 300 µl MeOH to allow 80:20 v/v mixture of high purity MeOH:water. The cells were disrupted using an ultrasound sonicator (Qsonica, CT, USA) for 10–20 s until no more intact cell could be detected. The total protein content of the homogenates was quantified according to the manufacturer’s protocol of the Bradford assay (Bio Rad) to control for potential differences in tissue quantities. After being stored at − 20 ℃ for at least 2 h to precipitate the proteins, the tubes were centrifuged at 14,000 rcf for 10 min at 4 ℃. The supernatant was transferred to a new Eppendorf tube and evaporated to dryness at room temperature in a speedvac concentrator (Savant, Thermo Fisher Scientific). The dried samples were reconstituted in 100 µl of 60% MeOH with 0.1% formic acid (FA) (Sigma-Aldrich) and transferred to plastic vials for LC–MS measurements.

Metabolite separation was achieved using an Agilent 1260 series HPLC system (Agilent, Santa Clara, CA). The injection volume of each sample was 5 µL and the column was maintained at 35 °C. QCs (pool of all samples within the experiment) and standards were run at the beginning and the end of each sequence and every four sample runs to monitor shift in the retention time on the column. For negative mode, a Cogent Diamond Hydride TM (MicroSolv, Eatontown, NJ, USA, Cat# 70,000-15P-2) aqueous normal phase (ANP) column (150 × 2.1 mm i.d., 4 µm particle size, 100 Å pore size) was used. The run time was 25 min at a flow rate of 0.4 ml/min. Chromatography was performed using solvent A (50% MeOH/50% water/0.05% FA) and solvent B (90% acetonitrile with 5 mM ammonium acetate). The gradient was: 0 min, 100% B; 20–25 min, 40% B; post-run time for equilibration, 10 min in 100% B. For positive mode, a Targa C18 reverse phase column (50 × 2.1 mm i.d., 3 µm particle size, 120 Å pore size, Higgens Analytical Mountain View, CA, USA, Cat# TS-0521-C183) was used. The run time was 25 min at a flow rate of 0.3 ml/min. Chromatography was performed using solvent A (Water with 0.1% formic acid) and solvent B (98% acetonitrile/2% water with 0.1% formic acid). The gradient was: 0 min, 2% B; 20–25 min, 100% B; post-run time for equilibration, 5 min in 2% B.

A 6520B Q-TOF LC–MS (Agilent, Santa Clara, CA) was operated in both positive and negative electrospray ionization (ESI) modes with an acquisition rate of 1.5 spectra/s in extended dynamic range (1700 *m/z*, 2 GHz). The spectra were internally mass calibrated in real time by continuous infusion of a reference mass solution using an isocratic pump connected to a dual sprayer feeding into an electrospray ionization source. Data were acquired with MassHunter Acquisition software B.05.01 and further processed with Agilent Profinder B.09 (Agilent, Santa Clara, CA).

The instrument settings were as follows: ion polarity, negative; gas temperature, 325 °C; drying gas, 10 l/min; nebulizer pressure, 45 psi; capillary voltage, 4000 V; fragmentor, 140 V; skimmer, 65 V; mass range, 70–1100 m/z; ion polarity, positive; gas temperature, 325 °C; drying gas, 10 l/min; nebulizer pressure, 45 psi; capillary voltage, 4000 V; fragmentor, 140 V; skimmer, 65 V.

For the data processing and chemometric analysis of the LC–MS untargeted data, the acquired raw data files (.d files) were first checked for quality in MassHunter Qualitative Analysis software (Agilent, version 7.0). Reproducibility of chromatograms was visually inspected by overlaying the total ion chromatograms (TICs) of all samples. Data files that exhibit outlier peaks, i.e., replicates with very dissimilar chromatograms (e.g., significant retention time shifts), were excluded for further processing. The raw data files were then converted to mzXML using ProteoWizard 3.0 (Kessner et al. 2008). Raw LC–MS data were analyzed by the MZmine 2 software (Pluskal et al. 2010) for chromatogram deconvolution, peak detection and alignment. The metabolites were called by batch-targeted feature extraction. The putative identification was achieved by online searching for the accurate *m/z* values of the peaks against HMDB and KEGG databases (Kanehisa and Goto 2000; Wishart et al. 2018). Those peaks were manually inspected for the quality of the extracted ion chromatograms (plausible adduct formation, max mass deviation 5 ppm, isotope ratios and peak shape) and for the remaining duplicate compound names. Data were calculated and presented as average log_2_-fold change in each independent experiment ± SD of at least two independent experiments performed in four to six replicates. Differences between treated and non-treated groups were assessed by two-way ANOVA (GraphPad Prism 8.4.3), followed by Bonferroni’s comparison post hoc test including correction for multiple testing. Post hoc test was only performed vs. controls. Statistical significance is indicated as follows: *p < 0.05.

## Results

To characterize the metabolic perturbation of FR, 3D rat primary neural cell cultures were obtained from 16-day-old fetal rat brains and were treated with FR from *DIV7* until *DIV*14 or *DIV* 21 to cover critical periods of differentiation and maturation. The potential DNT effects of FR were assessed through the expression of specific genes selected from a previous work (Hogberg et al. 2009,2010) that serve as markers for the structural and functional development during subsequent stages of neuronal and glial differentiation. Additional genes were selected based on IPP microarray data. Furthermore, untargeted metabolomics was performed using a quadrupole time-of-flight liquid chromatography mass spectrometry (Q-TOF LC–MS). Metabolomics involves the analysis of metabolic profiles in living cells in response to physiological alterations triggered by endogenous or exogenous elements, such as chemicals and pathological and developmental factors (Nicholson et al. 2012; van Vliet et al. 2013, 2008).

### Assessment of cell viability of 3D rat primary neural cell cultures exposed to FR

**Fig. 2:**
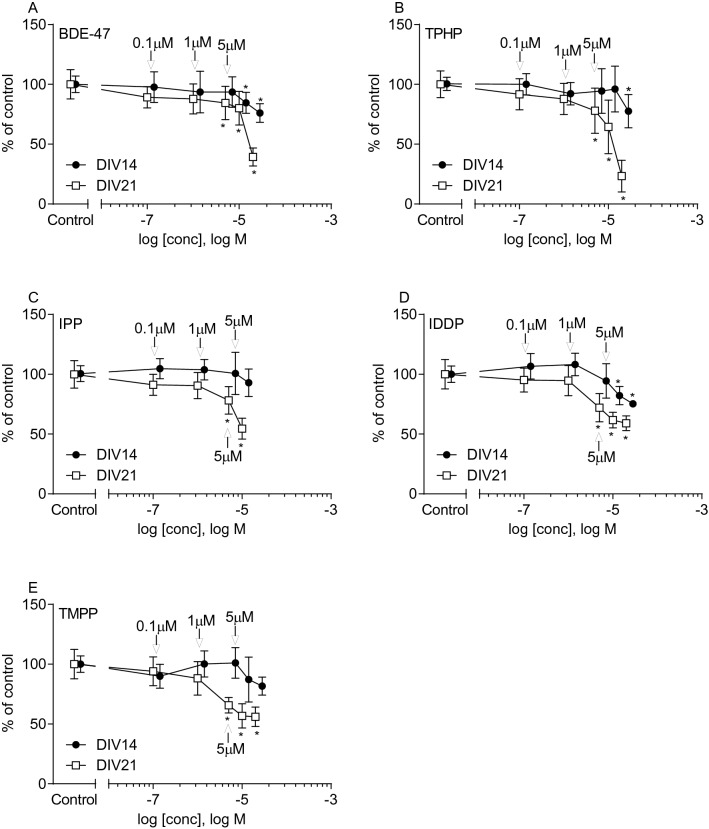
Cell viability at *DIV*14 (filled circle) and 21 (open square) using resazurin reduction assay after exposure at 7*DIV* to FR **a** BDE-47, **b** TPHP, **c** IPP, **d** IDDP and **e** TMPP. All data were normalized to mean of untreated control samples (100%) and are presented as mean ± SD of at least three independent experiments performed in 2–4 replicates. Differences between treated and non-treated (control) groups were assessed by one-way ANOVA (GraphPad Prism 8.4.3), followed by Dunnett’s multiple comparison post hoc test including correction for multiple testing. Post hoc test was only performed vs. controls. Statistical significance is indicated as follow *p < 0.05 (treated *vs.* control). Arrows indicate concentrations selected for metabolomics and transcriptomics experiments (0.1, 1 and 5 µM)

To determine non-cytotoxic concentrations of FR in rat brainspheres, aggregates were exposed to FR for 7 or 14 days starting from *DIV*7. Cytotoxicity was assessed using the resazurin cell viability assay at two different time points—*DIV*14 and *DIV*21. Initially, a wide range of concentrations for each FR was tested (Fig. 2, Table 2). Time and dose-dependent decrease in cell viability was induced by all FR (*DIV*14 vs*. DIV*21). At *DIV*14, 7 day exposure to 10 µM of BDE-47 (Fig. 2a), IDDP (Fig. 2d) and TMPP (Fig. 2e) induced significant reduction of the cell viability, while TPHP (Fig. 2B) only showed significant decrease at 20 µM. IPP (Fig. 2c) was only tested up to 10 µM where no significant effect was observed at *DIV*14. At *DIV*21*,* 14 days exposure to 5 µM of all FR showed a significant decrease in cell viability (Fig. 2 and Table 2). The level of cytotoxicity caused by the FR was used as reference for the selection of concentrations for gene expression and metabolomics analyses. In conclusion, 0.1 µM, 1 µM (non-cytotoxic at 14 and 21 *DIV*) and 5 µM (non-cytotoxic at 14 *DIV* and lowest observed cytotoxicity effect at 21 *DIV*, ~ 70–80% viability vs*.* control) were selected for further experiments.

**Table 2:** Cell viability (resazurin assay) after exposure to FR

Conc. (µM)	BDE-47	TPHP	IPP	IDDP	TMPP
*DIV*14 (% of control)					
Control	100 ± 6.9	100 ± 5.5	100 ± 6.3	100 ± 6.9	100 ± 6.9
0.1	97.7 ± 12.7	100.0 ± 8.9	104.6 ± 8.4	106.7 ± 10.6	89.9 ± 10.1
1	93.6 ± 17.4	92.2 ± 9.5	103.8 ± 8.5	108.2 ± 9.4	100.2 ± 10.9
5	93.6 ± 12.7	94.4 ± 18.7	100.7 ± 17.6	94.4 ± 14.3	101.1 ± 12.8
10	84.5 ± 8.8*	96.1 ± 19.2	92.9 ± 11.5	82.2 ± 7.6*	87.2 ± 18.8
20	76.0 ± 7.8*	77.5 ± 13.9*		75.3 ± 0.6*	81.7 ± 7.5
*DIV*21 (% of control)					
Control	100 ± 12.3	100 ± 11.2	100 ± 11.5	100 ± 12.3	100 ± 12.3
0.1	89.2 ± 8.9	91.6 ± 13.1	91.2 ± 8.7	95.2 ± 10.0	94.1 ± 12.1
1	87.8 ± 12.5	87.9 ± 13.1	90.6 ± 10.9	94.7 ± 12.7	88.1 ± 14.0
5	84.4 ± 13.9*	77.9 ± 18.9*	78.2 ± 11.5*	72.1 ± 11.8*	65.6 ± 6.5*
10	80.0 ± 14.1*	64.4 ± 22.4*	54.5 ± 8.8*	61.7 ± 6.6*	56.8 ± 10.1*
20	39.3 ± 7.5*	23.3 ± 13.3*		59.0 ± 6.3*	56.0 ± 8.2*

Data expressed as mean ± SD. Statistical significance is indcated as follow *p < 0.05 (treated *vs.* control), using one-way ANOVA, Dunnett’s Multiple Comparison Test. followed by Dunnett’s multiple comparison post hoc test including correction for multiple testing. Post hoc test was only performed vs. controls

### Exposure to FR significantly altered marker genes involved in neuronal morphology and function

**Fig. 3:**
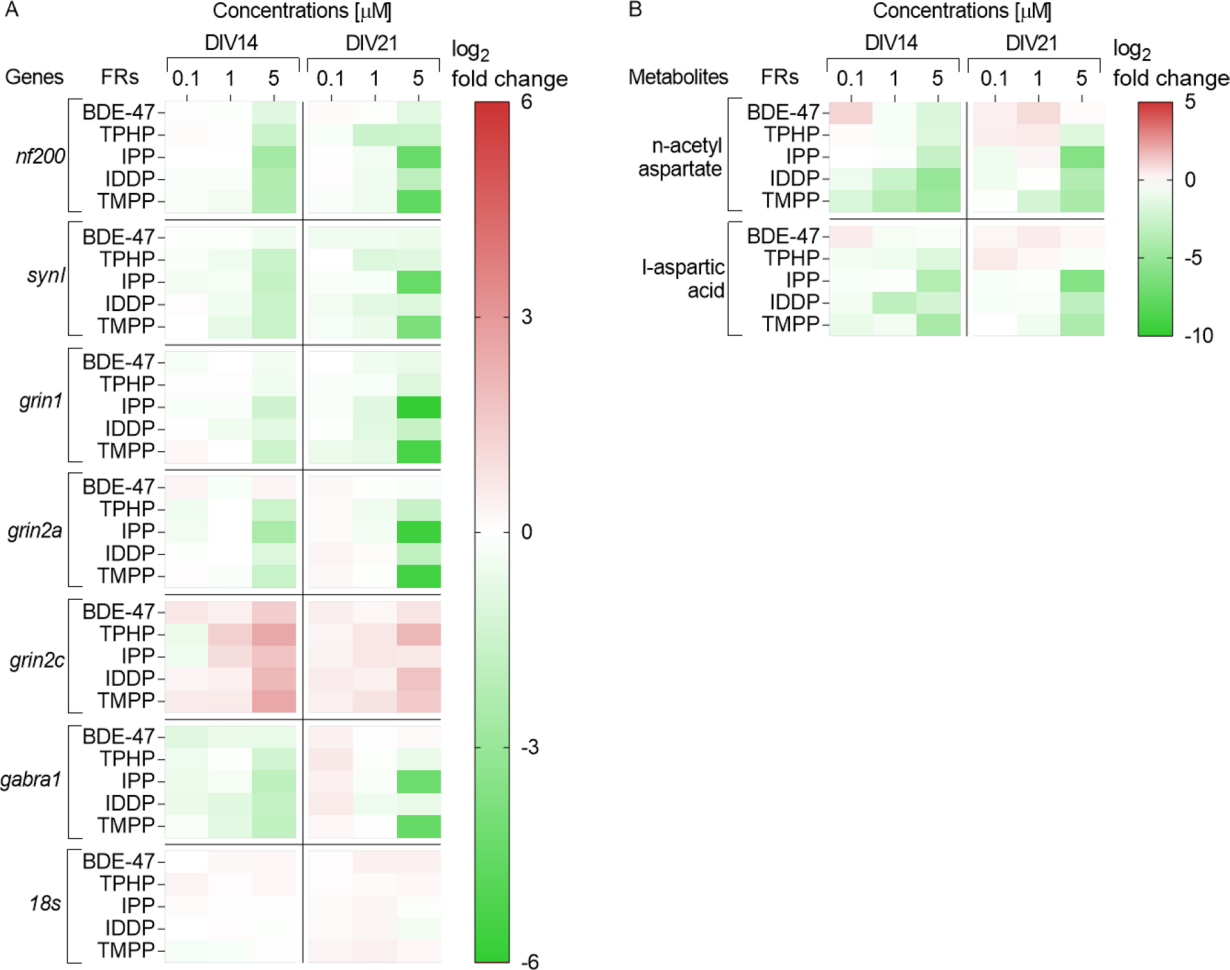
Heatmap (double gradient, green—minus; red—plus) illustrating (A) genes (measured with RT-qPCR) and (B) metabolites (measured with Q-TOF LC–MS), involved in neuronal morphology and function after exposure to FR. All data were normalized to mean of untreated control cells (0) and are displayed as means log_2_-fold change in each independent experiment, from at least three independent experiments performed in 2–3 replicates (genes) or at least two independent experiments performed in 4–6 replicates (metabolites)

To assess FR effects on neurons, neurofilament 200 (*nf-200*) as an intermediate filament highly expressed in neurons during the later stages of differentiation was chosen. It is an important cytoskeleton marker, whose expression can be used to detect neuronal morphology changes (Gupta et al. 1999; Tonnaer et al. 2010). The mRNA levels of *nf-200* were significantly downregulated after exposure to all FR (5 µM), at *DIV*14 (no cytotoxicity detected) (Fig. 3a and suppl. material 1). Further decrease was observed at *DIV*21 after exposure to 5 µM IPP and TMPP and already 1 µM TPHP exposure significantly decreased the mRNA expression of *nf-20*0 at *DIV*21 (no cytotoxicity observed). In conclusion, all FR studies were toxic to neurons in the low µM range.

The mRNA expression of two receptors that play crucial roles in neuronal function were addressed to further assess neuronal impairment: subunits (*grin1*, *grin2a* and *grin2c*) of the ionotropic N-methyl D-aspartate receptor (NMDA-R) of the main excitatory neurotransmitter glutamate, (Blanke and VanDongen 2009; Busse et al. 2014) and subunit alpha 1 (*gabra1*) of the main inhibitory neurotransmitter gamma-aminobutyric acid A receptor (GABA_A_-R) (Sigel and Steinmann 2012).

Exposure to the highest concentration (5 µM) of IPP, IDDP and TMPP significantly decreased mRNA levels of the NMDA-R subunit *grin1* at *DIV*14 and was further decreased at *DIV*21 (Fig. 3a and suppl. material 1), although at *DIV*21, already the lower concentration (1 µM) of these FR (IPP, IDDP and TMPP) downregulated the expression of *grin1*. Exposure to the highest concentration (5 µM) also decreased the mRNA levels of *grin1* at 21 *DIV* for BDE-47, and TPHP. The gene expression of the NMDA-R subunit *grin2a* was significantly downregulated after exposure to all OPFR both at 14 and 21 *DIV* (Fig. 3a and suppl. material 1). Exposure to BDE-47 did not alter the expression of *grin2a.* In contrast, the expression of subunit *grin2c* was significantly upregulated after exposure to all FR (5 µM) both at *DIV*14 and *DIV*21 (Fig. 3a and suppl. material 1). Already the lower concentration of 1 µM increased the mRNA levels after exposure to TPHP, IPP and TMPP at 14 and/or 21 *DIV*) (Fig. 3a and suppl. material 1). In conclusion, with slight differences in active concentrations, all FR decreased *grin1*and *grin2a*, but increased *grin2c* expression that could affect the affinity of the receptor.

The mRNA expression of GABA_A_-R subunit alpha 1 (*gabra1*) was significantly downregulated after exposure to 5 µM of all OPFR at 14 *DIV* (Fig. 3a and suppl. material 1). Already exposure to 1 µM of IDDP and TMPP significantly decreased the mRNA level of *gabra1* at *DIV*14. After longer treatment (*DIV*21) only exposure to IPP and TMPP (5 µM) continued to decrease in the mRNA level of *gabra1*, while TPHP and IDDP treated cultures were reversed to control levels. Exposure to BDE-47 did not alter the expression of *gabra1*. In conclusion, the replacement FRs downregulated *gabra1*, while the same concentration of BDE-47 had no effect*.*

Furthermore, the expression of the neuronal marker synapsin 1 (*syn1*) was evaluated. SYN1 is a protein present in the membrane of synaptic vesicles (Lu et al. 1992). It modulates neurotransmitter release and is believed to exert an important role in the functional maturation of synapses (Harrill et al. 2011). Already the lower concentration of 1 µM decreased the expression of *syn1* for most of the OPFR at *DIV*14 and *DIV*21 (Fig. 3a and suppl. material 1). Exposure to 5 µM of all the OPFR downregulated the expression of *syn1* at both time points. BDE-47 did not have any effect on the expression of *syn1* at those concentrations. In conclusion, the expression of *syn1* was significantly downregulated after exposure to OPFRs, but not to BDE-47.

To characterize the perturbation of brainspheres on metabolic level, the neural-specific metabolite n-acetyl aspartate (NAA) was measured. NAA is considered a diagnostic molecule for patients with brain damage and neurodegenerative disorders (Alakkas et al. 2019; Baslow et al. 2003; Chitturi et al. 2018; Nordengen et al. 2015). Mass spectrometry revealed that NAA was significantly lower after exposure to IPP (5 µM), IDDP (1 and 5 µM) and TMPP (1 and 5 µM) at 14 and 21 *DIV* (Fig. 3b and suppl. material 1). l-Aspartic acid, the precursor of NAA, was also significantly lower after exposure to all OPFRs (Fig. 3b and suppl. material 1). This indicates that OPFRs induce toxicity in rat brainspheres.

In summary, the exposure to OPFR induced stronger effects on NAA, l-aspartic acid and selected genes involved in neuronal morphology and function than BDE-47. In addition, expression of subunits of the NMDA-R were more affected by exposure to OPFR than the subunit of the GABA_A_-R.

### Exposure to OPFR significantly decreased the level of neurotransmitters and downregulated the expression of genes involved in their production and transportation

**Fig. 4:**
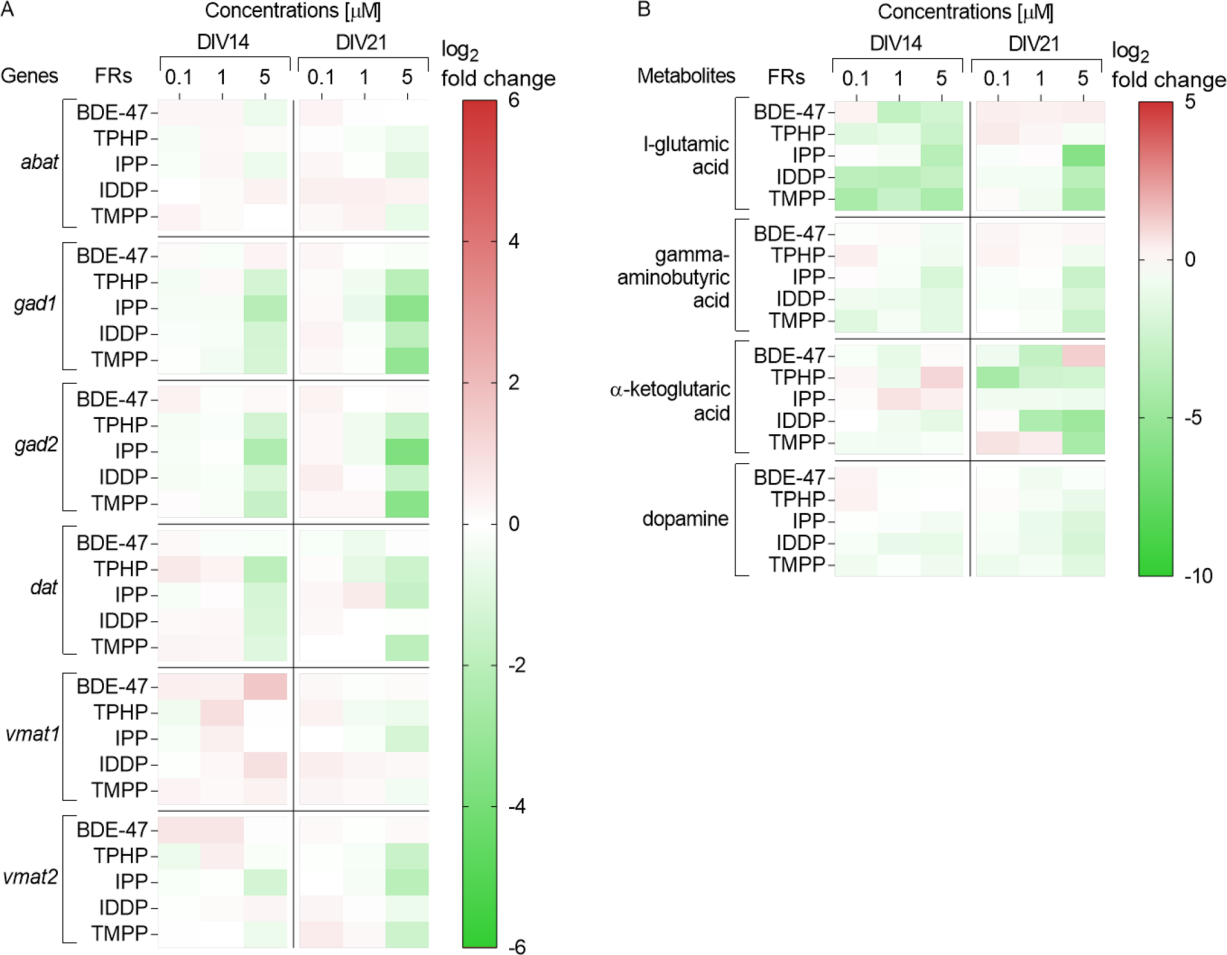
Heatmap (double gradient, green—minus; red—plus) illustrating **a** genes (measured with RT-qPCR) and **b** neurotransmitters (metabolites) (measured with Q-TOF LC–MS), involved in neurotransmitter transportation and production after exposure to FR. All data were normalized to mean of untreated control cells (0) and are displayed as means log_2_-fold change in each independent experiment, from at least three independent experiments performed in 2–3 replicates (genes) or at least two independent experiments performed in 4–6 replicates (metabolites)

To further understand the effect of FR on neuronal cells, we evaluated expression of the enzymes catalyzing neurotransmitter production and neurotransmitter transporters. Neurotransmitters GABA and glutamate (l-glutamic acid) play important regulatory roles in neuronal activities in the brain (Busse et al. 2014; Sigel and Steinmann 2012). Therefore, the expression of genes encoding two enzymes, glutamate decarboxylase *gad1* and *gad2* that catalyze the synthesis of GABA from glutamate and the key enzyme responsible for catalyzing GABA degradation, 4-aminobutyrate transaminase (*abat*), was investigated. Treatment with 5 µM of all OPFRs, but not BDE-47, significantly decreased the expression of *gad1* and *gad2* at both *DIV*14 and *DIV*21 (Fig. 4A and suppl. material 1). The expression of *abat* was only affected by exposure to 5 µM IPP at *DIV*21.

Metabolomics analysis showed a significant decrease in the neurotransmitter glutamate at *DIV*14 after exposure to all FR (Fig. 4b and suppl. material 1). The strongest effect was observed after exposure to IDDP and TMPP as already the lowest concentration of 0.1 µM was effective. After *DIV*21, the amount of glutamate was restored except for the highest concentration of IPP, IDDP and TMPP (5 µM). Similar effects were observed on GABA: exposure to IDDP and TMPP decreased levels of GABA already at the lowest concentration (0.1 µM) and was restored at *DIV*21 except for 5 µM of IPP, IDDP and TMPP (Fig. 4b and suppl. material 1). Exposure to BDE-47 and TPHP did not alter the levels of GABA. Moreover, α-ketoglutaric acid a derivate of glutamate were significantly decreased at *DIV*21 after exposure to all FR except IPP, with TPHP having the strongest effect (Fig. 4b and suppl. material 1). In conclusion, all FR decreased the neurotransmitters glutamate, GABA (except BDE-47 and TPHP) and α-ketoglutaric acid (except IPP).

Next, we addressed the dopaminergic neurotransmitter system. The plasma membrane dopamine active transporter (*dat—slc6a3*) and the vesicular monoamine transporter (*vmat1—slc18a1* and *vmat2—slc18a2*) are critical components for dopamine neurotransmission: The plasma membrane dopamine active transporter terminates the signal of the neurotransmitter by providing dopamine reuptake from the synaptic cleft, while vesicular monoamine transporter packages cytoplasmic dopamine into vesicles for storage and future use (Miller et al. 1999; Yamamoto et al. 2007). Exposure to all OPFR (5 µM) at *DIV*14 decreased the mRNA expression of *dat* (Fig. 4A and suppl. material 1). At *DIV*21, the expression of *dat* after exposure to IDDP was returned to control levels, while the decrease was similar *to DIV*14 levels for TPHP, IPP and TMPP. BDE-47 treatment did not modify the gene expression of *dat*. In conclusion, at similar concentrations, the FR currently in use decreased plasma membrane dopamine active transporter expression, while BDE-47 did not.

Treatment with OPFRs did not alter either *vmat1* or *vmat2* mRNA levels at *DIV*14 (Fig. 4a and suppl. material 1). Interestingly, treatment with BDE-47, at *DIV*14 significantly increased the *vmat1* gene expression. At 21 *DIV* significant downregulation of *vmat1* gene expression was observed only after exposure to 5 µM IPP, while *vmat2* mRNA expression was significantly downregulated after exposure to 5 µM IPP, TMPP and TPHP. BDE-47 did not affect the expression of either transporter at *DIV*21. Metabolomics analysis showed a significant decrease in the neurotransmitter dopamine after exposure to all OPFRs, but not BDE-47. Again, exposure to IDDP and TMPP had the strongest effect, as the decrease was observed already at *DIV*14 (Fig. 4b and suppl. material 1). In conclusion, BDE-47 showed very different effects to the other FR increasing not decreasing *vmat1*and no effect on *vmat2* expression.

In summary, genes involved in the enzymatic transformation of glutamate to GABA were affected by all FRs at non-cytotoxic concentrations, again with the strongest effects observed by exposure to OPFRs. This indicates that the ratio of these neurotransmitters might be altered. In fact, the neurotransmitters glutamate and GABA were decreased as well as α-ketoglutaric acid (glutamate derivate). In addition, the expression of the dopaminergic transporter *dat* and dopamine levels were decreased for all OPFRs. Less prominent effects were observed on genes encoding the vesicular transporters and only at the highest concentrations. Minimal effects were observed after exposure to BDE-47 on selected genes.

### Exposure to FR affects the gene expression of glial markers

One advantage of the 3D rat primary neural cell cultures is the presence of different cell types of the brain including glial cells, (astrocytes, oligodendrocytes and microglia). Aiming to assess glial toxicity due to exposure to FR, the gene expression of two specific markers for mature astrocytes were evaluated: glial fibrillary acidic protein (*gfap*) and the calcium-zinc-binding protein s100 beta (*s100β*).

**Fig. 5:**
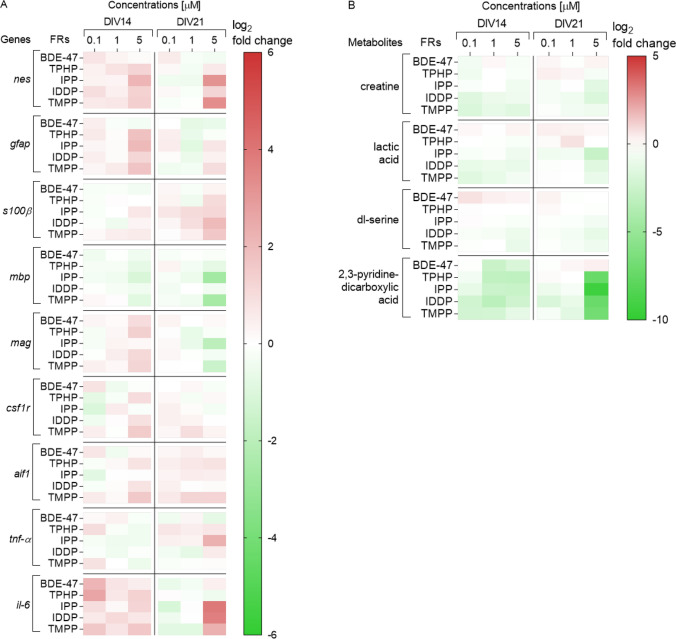
Heatmap (double gradient, green—minus; red—plus) illustrating **a** genes (measured with RT-qPCR) and **b** metabolites (measured with Q-TOF LC–MS), identified in glial cells (astrocytes, oligodendrocytes and microglia) after exposure to FR. All data were normalized to mean of untreated control cells (0) and are displayed as means log_2_-fold change in each independent experiment, from at least three independent experiments performed in 2–3 replicates (genes) or at least two independent experiments performed in 4–6 replicates (metabolites)

The expression of *gfap* was upregulated after the exposure to all OPFR (5 µM) at *DIV*14 (Fig. 5a and suppl. material 1). However, this effect was lower or even reversed at *DIV*21. OPFR exposure altered the mRNA levels of the astrocytic marker *s100β* at a later stage with significant increase at *DIV*21. On the contrary, BDE-47 decreased *gfap* expression at 21 *DIV*. This might indicate an astrogliosis induced by the OPFR not seen for BDE-47.

The effect of FR on the initial stages of the CNS developmental process, including proliferation of progenitor cells, was assessed by the expression of *nestin*. This protein is a cytoskeletal intermediate filament that is gradually replaced by cell-specific intermediate filaments such as nf-68 and nf-200 in the neural cells and gfap in astrocytes during the course of the CNS differentiation and maturation (Jin et al. 2009; Wiese et al. 2004). Furthermore, nestin can be re-expressed in activated astrocytes in the event of brain or neuronal injury and for this reason it is also recognized as a sensitive marker for reactive astrocytes (Brook et al. 1999; Chen et al. 2002; Sergent-Tanguy et al. 2006).

*Nestin* expression at *DIV*14 was significantly upregulated at the highest concentration (5 µM) and increased even further at *DIV*21 after exposure to IPP, IDDP and TMPP and was not affected by the exposure to BDE-47 and TPHP at *DIV*21 (Fig. 5a and suppl. material 1). This is concordant with astrogliosis induced by the OPFR, but not BDE-47.

Lactic acid is mainly produced in astrocytes in the CNS and is an essential element of neuron–glia metabolic interactions (Pellerin 2003). We observed a decrease in lactic acid after exposure to IPP, IDDP and TMPP (Fig. 5b and suppl. material 1). Creatine is a metabolite that has shown to be a marker of gliosis when increased (Konaka et al. 2003) as well as a marker of neurotoxicity when decreased (Diserens et al. 2018; van Vliet et al. 2008). Exposure to IPP, IDDP and TMPP significantly decreased the levels of creatine (Fig. 5b and suppl. material 1). Moreover, a significant decrease in dl-serine was observed after exposure to IPP, IDDP and TMPP with stronger effects to IDDP and TMPP (Fig. 5b and suppl. material 1). D-serine is considered an astroglia-derived neurotransmitter (Van Horn et al. 2013), though it is also produced marginally in neurons. However, with the method chosen here for mass spectrometry, it is not possible to distinguish between d- and l-isoforms, therefore, they are reported together.

In summary, expression of glial markers indicates that exposure to OPFR may activate astrocytes, either in a primary or secondary fashion, due to neuronal damage while less effect was observed after exposure to BDE-47 on selected genes and metabolites.

Myelin basic protein (*mbp*) is a protein expressed in mature oligodendrocytes and is a well-recognized oligodendrocytic marker. The highest concentration (5 µM) of TPHP, IPP and TMPP significantly downregulated *mbp* expression at both *DIV*14 and *DIV*21 (Fig. 5a and suppl. material 1). Exposure to BDE-47 and IDDP did not interfere with the expression of *mbp* mRNA. In conclusion, at the higher concentration three of the OPFR but not BDE-47 might interfere with myelination.

To further analyze the possible effects on myelination, myelin-associated glycoprotein (*mag*) was assessed. The *mag* is a transmembrane glycoprotein essential for the formation and maintenance of the myelin sheath by promoting glia–axon interactions (Paivalainen and Heape 2007; Quarles 2007). At *DIV*14, the expression of *mag* was upregulated by exposure to all FR (5 µM) except IPP (Fig. 5a and suppl. material 1). At *DIV*21, the expression was instead significantly downregulated by IPP and TMPP (5 µM) and back to control levels in BDE-47, TPHP- and IDDP-treated samples. This indicates some possible restored effects on myelination by these FR.

### Exposure to FR may trigger an inflammatory response

The inflammatory cytokines interleukin-6 (IL-6) and tumor necrosis factor-alpha (TNF-α) are synthesized by cells of the CNS, including neurons and glial cells, to exert neuroprotective roles (Carlson et al. 1999). A significant increase in the expression of *il-6* was observed after exposure to the highest concentration (5 µM) of IPP, IDDP and TMPP at *DIV*21 (Fig. 5A and suppl. material 1). No change was observed after exposure to BDE-47 and TPHP. Expression of *tnf-α* was significantly increased only after exposure to 5 µM IPP at *DIV*21 (Fig. 5a and suppl. material 1).

To characterize further the inflammatory component, the macrophage colony-stimulating factor 1 receptor (*csf1r*), involved in microglial proliferation and activation (Mitrasinovic et al. 2005), and the allograft inflammatory factor (*aif*), upregulated in activated microglia due to inflammation (Deininger et al. 2002), were evaluated. The expression of *csf1r* and *aif* was only increased after exposure to TMPP (Fig. 5a and suppl. material 1). In contrast, levels of the metabolite 2,3-pyridinedicarboxylic acid (quinolinic acid) produced by activated microglia was significantly decreased after exposure to all OPFRs (Fig. 5b and suppl. material 1).

The increase of cytokine gene expression indicates that exposure to OPFRs may induce an inflammatory response in the rat primary neural model, but the microglia are only activated after exposure to TMPP. The latter was also supported by the metabolite observation. However, to fully confirm the lack of activated microglia, additional time course studies need to be performed.

### Exposure to IPP modified the transcriptome

To get a more complete representation and to identify additional genes and pathways of interest, the whole transcriptome was analyzed after exposure to 1 µM IPP at 21*DIV* versus correspondent solvent control. We observed 1971 significantly different expressed genes (|FC|> 1.4 and *p* < 0.05 false discovery rate Benjamini–Hochberg procedure), which were further analyzed by pathway/category overrepresentation analysis using KEGG, WikiPathways and Reactome (Fabregat et al. 2018; Kanehisa and Goto 2000). The top perturbed pathways were associated with four main mechanisms by overrepresentation gene analyses (Table 3):

1. downregulation of neurotransmitter receptors including VMAT1 and associated intracellular signal transduction (G-protein, Ca^2+^, MAPK);
2. upregulation of immune response, inflammation and defense response mainly driven by MHC-I, FasL, Fas, complement system, FC IgG receptors (macrophages) and AIF (specific microglial activation biomarker);
3. upregulation of cell cycle;
4. changed fatty acid metabolism and transportation.

**Table 3:** Whole transcriptome analyzes using KEGG, WikiPathways and Reactome after exposure to 1 µM IPP at 21*DIV* revealed alternations in genes belonging to four main mechanism, (1) neurotransmitters, (2) immune response, cell cycle and (4) fatty acid metabolism and transportation

Group	KEGG, WikiPathways, Reactome	*p*-value (uncorrected)	Matched	Total	Interpretation
1	Alcoholism	< 1E−12	46	175	_↓_ VMAT1, postsynaptic neurotransmitter receptors, intracellular signal transduction
2,3	Systemic lupus erythematosus	3.40E−12	36	126	_↑_ Complement cascades, membrane attacking complex, FC IgG receptors (macrophages), histones
1	Neuroactive ligand-receptor interaction	7.93E−07	47	289	_↓_ Neurotransmitter receptors: 1.amine: acetylcholine, epinephrine, 5-HT 2.peptides: angiotensin, Lipoxin A4, cholecystokinin, opioids, orexin, somastatin, tachykinin 3.seretin like: vasoactive intesinal peptide, 4.others: glutamate, GABA, nucleotides, aspartate, cystein, glycine, alanine, growth hormone ↑ Neurotransmitter receptors: 1.amine: trace amine 2.peptide: neuropeptide Y 3.prostanoid (local inflammation) 4.class B secretin like: vasoactive intestinal peptide
1	Calcium signaling pathway	1.54E−06	34	183	_↓_ Neurotransmitter receptors, calcium signaling proteins—> leads to MAPK signaling pathways
1,2,3	Viral carcinogenesis	2.16E−05	36	234	_↓_ MAPK, ↑ p53, PI3K, cell cycle, MHC-I
4	Rn_Statin_Pathway_WP145_79447	2.90E−05	8	19	
1	Rn_GPCRs,_Class_A_Rhodopsin-like_WP473_72158	3.52E−05	36	229	_↓_ Neurotransmitter receptors
	Rn_D-Glucose-Ins1-Rxra_WP2043_71283	4.65E−05	9	25	
4	Biosynthesis of unsaturated fatty acids	1.35E−04	9	27	
1	Rn_Monoamine_GPCRs_WP276_48267	1.42E−04	10	33	_↓_ Neurotransmitter receptors
	Rn_Endochondral_Ossification_WP1308_72214	1.66E−04	14	61	
4	PPAR signaling pathway	2.91E−04	16	78	Lipids metabolism and transportation
3	Rn_Cell_cycle_WP429_41778	7.93−04	16	88	_↓↑_ Cyclins D, A, B
4	Rn_Cholesterol_metabolism_WP632_77527	1.39E−03	7	23	
1	Rn_Peptide_GPCRs_WP131_71770	2.06E−03	13	69	_↓_ Neurotransmitter receptors
4	Rn_Adipogenesis_WP155_41714	2.15E−03	20	130	Fatty acid metabolism and transportation
1,2	Cell adhesion molecules (CAMs)	2.44E−03	24	172	_↑_ MHC-I, Myelin associated glycoprotein, ↓ neuronreceptors, epithelium cells receptors
1	Cocaine addiction	2.91E−03	10	47	_↓_ VMAT1, postsynaptic neurotransmitter receptors, intracellular signal transduction
1,2	Type I diabetes mellitus	3.07E−03	13	75	_↓_ Glutamate metabolism, ↑ MHC-I
1	Alanine, aspartate and glutamate metabolism	3.90E−03	8	36	_↓_ Peptide neurotransmitter metabolism
1	Amphetamine addiction	4.07E−03	12	65	_↓_ VMAT1, postsynaptic neurotransmitter receptors, intracellular signal transduction
	Rn_Spinal_Cord_Injury_WP2433_78470	4.07E−03	16	102	
1	Rn_GPCRs,_Other_WP409_41752	4.45E−03	13	75	_↓_ Neurotransmitter receptors
	Taurine and hypotaurine metabolism	5.28E−03	4	10	
	Rn_SIDS_Susceptibility_Pathways_WP1304_41670	5.39E−03	11	60	
	Rn_Cardiovascular_Signaling_WP590_41785	6.74E−03	8	38	
1	Vascular smooth muscle contraction	7.02E−03	18	124	_↓_ Neurotransmitter receptors
2	Graft-versus-host disease	7.91E−03	11	66	No toll-like receptor, TNFa, IL-1 deregulation but ↑ of FasL and Fas and MHC-I
2	Autoimmune thyroid disease	8.40E−03	12	76	_↑_ of FasL and Fas and MHC-I

The transcriptome and RT-PCR analysis indicates similar effects.

In conclusion, after gene-wise careful evaluation, most pathways/categories could be classified into four categories: (1) transmission of action potential, cell–cell signaling, synaptic transmission, receptor signaling, (2) immune response, inflammation, defense response, (3) cell cycle and (4) lipids metabolism and transportation.

## Discussion

The U.S. Consumer Product Safety Commission (CPSC) accepted a petition to ban furniture, children’s products, electronic enclosures, and mattresses containing any member of the class of organohalogen flame retardants in 2017. In response to the petition, the National Academies of Sciences (NAS) released a report in May 2019, titled *“Scoping Report for Conducting a Hazard Assessment of Organohalogen Flame Retardants as a Class”* (National Academies of Sciences 2019). The authors concluded that there is a need to categorize flame retardants into broad classes for risk assessment rather than regulate on individual compounds.

Following the voluntary phase-out of the PBDEs, there has already been an increase in the use of the OPFR as evidenced by their ubiquitous detection in air and in dust in a variety of indoor environments (Bergh et al. 2011; Hoffman et al. 2015; Stapleton et al. 2009). A recent study showed the presence of OPFRs in children from residential exposures (Phillips et al. 2018). OPFR metabolites have also been detected in urine in the general population (Hammel et al. 2016; Hoffman et al. 2017; Ospina et al. 2018). Considering the recent petition on the ban on organohalogens, the use of OPFR is further projected to be on the rise. However, their toxicological hazard has not yet been well characterized. Several recent streams of data are converging on the conclusion that OPFRs show equivalent (or in some cases greater) toxicity compared to some of the phased-out PBDEs in several human-derived cell-based models (Behl et al. 2015). These compounds have also been shown to produce neurobehavioral deficits in complementary animal models (Alzualde et al. 2018; Bailey and Levin 2015; Glazer et al. 2018; Oliveri et al. 2015; Yan et al. 2017; Zhang et al. 2019) and might be a regretful substitution (Zimmerman and Anastas 2015).

The current study sheds light on possible mechanisms by which OPFR may exert their developmental neurotoxicity using a 3D primary rat neural culture. The aggregates were exposed to non- or low-cytotoxic concentrations of BDE-47, TPHP, IPP, IDDP and TMPP (0.1, 1 and 5 µM) to understand the toxicity mechanism and reveal potential perturbations to crucial neurodevelopmental processes, including neuronal and glial differentiation, and functional maturation. Metabolomics and gene expression were evaluated in control and FR-treated cultures at *DIV*14 and *DIV*21*,* after 7 and 14 days of exposure, respectively.

Exposure to FRs altered several markers involved in neuronal morphology and function, both at the gene and metabolome level. A decrease was observed in the mRNA levels of *nf-200*, an essential component of the axonal cytoskeleton. The synthesis of nf proteins and the timely suppression of *nf* gene expression are of functional importance for neuronal activity during the differentiation and maturation process of the CNS. Hence, nf-200, a late developing cytoskeleton protein, appears as a crucial element for neuronal function, since it is involved in the regulation of the axonal growth and is believed to provide nf stability and resistance to protein breakdown by retarding the slower axonal transport component (Goldstein et al. 1987; Liu et al. 1994). The decrease in the expression of neurofilaments after exposure to FRs indicates a structural disruption that can disturb the axonal organization and may cause degeneration of the axons (Perrone Capano et al. 2001) and the onset of neuropathology. Decreased expression of genes involved in cytoskeleton organization in neurite formation alongside with altered locomotor behavior has also been observed in zebrafish larvae after exposure to OPFR (Sun et al. 2016). Moreover, exposure to OPFR has previously been shown to affect neurite outgrowth at similar concentrations as in this study (Behl et al. 2015; Hausherr et al. 2014) supporting our results. Neurite outgrowth is one of the most established in vitro developmental neurotoxicity tests (Bal-Price et al. 2018) covering one of the key processes of CNS development that if perturbed likely leads to adverse outcomes.

Moreover, genes involved in neuronal function such as synaptogenesis and receptor expression were altered after exposure to all OPFR. Synapsin1 (syn1) has a functional role during neuronal development and playing an important part in the formation of synapses and in the regulation of neurotransmitter release by control of the amount of synaptic vesicles ready for exocytosis at the axon terminal (Evergren et al. 2007; Ferreira and Rapoport 2002). The decrease in the expression of *syn1* after exposure to OPFRs could impact the neuronal function and be linked to a defective neuron signaling system or even death. Previously, the expression of another synaptogenesis marker *syn2a* has been shown to be downregulated after exposure to OPFRs in zebrafish larvae (Sun et al. 2016) supporting disruption of neuronal signaling. Indeed, exposure to all FR used in this study has previously shown to decrease the neural network activity in rat primary cortical cultures acutely (Behl et al. 2015). Exposure to IPP and BDE-47 during development also showed decrease in activity in the network formation assay (NFA) (Shafer et al. 2019). However, exposure to TMPP, IDDP and TPHP did not induce any changes. The decreased activity (~ 6–16 µM) was reported close to the highest concentrations tested in the NFA (20 µM) and it is possible that a change in activity would be observed in the NFA if higher concentration had been tested. The decrease in *syn1* in the rat 3D model is clearly at lower concentrations (1 µM). The window of development, cell populations, exposure scenario and culture condition (2D vs. 3D) differ between these studies and could contribute to the vulnerability. Additional experiments need to be performed to understand if the effects observed in the rat 3D brainsphere model also leads to functional changes. Still, the decrease in levels of the metabolites NAA and L-aspartic acid observed in this study is a clear indication of neuronal damage and impaired neuronal function. NAA is a common biomarker used in patients to diagnose stroke and neurodegenerative disorders (Alakkas et al. 2019; Baslow et al. 2003) and is produced from l-aspartic acid and acetyl-coenzyme A (Ariyannur et al. 2010).

In the rat brainsphere model, we also observed that neurotransmitters and mRNA levels of their receptors were altered due to exposure to the selected FR. The strongest effect was noted in the expression of the glutamate NMDA receptor, where subunits *grin1* and *grin2a* were significantly downregulated at non-cytotoxic concentrations, especially after exposure to IPP and TMPP. The switch in subunit 2b–2a during development is crucial for proper maturation of the brain, as it is involved in neuronal function and synaptogenesis (Liu et al. 2004; Luthi et al. 2001). Interestingly, the expression of subunit *grin2c* was significantly increased after FR exposure and could be a sign of altered receptor affinity to the neurotransmitters (Yi et al. 2020). It was previously observed that exposure to TMPP reduces the response to glutamate in mouse cortical neurons (Hausherr et al. 2014). In this study, we observed a clear decrease in glutamate (l-glutamic acid) and the glutamate derivate α-ketoglutaric acid after the exposure to FR, which supports such theory. This was already observed at the lowest concentration (0.1 µM) and at the early time point (*DIV*14), indicating that this could be one of the mechanisms of the tested OPFR. Moreover, it is well documented that the NMDA receptor is a potential target during brain development and its inhibition can lead to decreased synaptogenesis, decreased neuronal network formation and ultimately impairment of learning and memory abilities (AOP 12, https://aopwiki.org/aops/12 and AOP 13, https://aopwiki.org/aops/13) (Sachana et al. 2018; Spinu et al. 2019; Wang et al. 2018). The altered gene expression of subunits of the NMDA receptor and the reduction in glutamate are therefore of high concern and of importance to investigate further.

GABA is the principal inhibiting neurotransmitter in the mature brain, but also exerts excitatory actions during the formation of the CNS (Ben-Ari et al. 2007; Li and Xu 2008). It is implicated in the proliferation of neural progenitor cells (Haydar et al. 2000), migration (Luhmann et al. 2015), differentiation (Barbin et al. 1993; Ganguly et al. 2001), outgrowth of neurites (Maric et al. 2001), and synaptogenesis (Ben-Ari 2002). The neurotransmitter GABA was decreased after exposure to IPP, IDDP and TMPP alongside with decreased expression of GABA_A_ receptor subunit alpha1 (*gabra1*), but to a lower degree than glutamate and its receptor and mainly after prolonged exposure (14 days). Moreover, two isoforms of glutamate decarboxylase (*gad1* and *gad2*), which are the enzymes responsible for catalyzing GABA synthesis from glutamate, were significantly decreased. This likely contributes to the decrease seen in GABA levels, but can also be a secondary effect due to the decrease in glutamate levels. Inhibition of the GABA_A_ receptor has previously been suggested to contribute to the toxicity of TMPP (Gant et al. 1987) and TPHP (Flaskos et al. 1998; Gant et al. 1987). Reduction of GABAergic neurons is one key event identified in AOPs (AOP 10, https://aopwiki.org/aops/10 and AOP 54 https://aopwiki.org/aops/54) for neurotoxicity and developmental neurotoxicity (Bal-Price et al. 2015b; Spinu et al. 2019; Westerholz et al. 2010) and could contribute to the toxicity of OPFR. Moreover, the ratio of glutamate/GABA has shown to be disturbed in autistic children (Gaetz et al. 2014; Gogolla et al. 2009; Rippon et al. 2007; Rubenstein and Merzenich 2003), raising a concern for OPFR as replacements, especially in children products, particularly as exposure to organophosphate pesticides and PBDEs is a suggested environmental risk factor for autism (Mostafalou and Abdollahi 2018; von Ehrenstein et al. 2019; Vuong et al. 2018; Ye et al. 2017).

Furthermore, decreases in the neurotransmitter dopamine and genes related to dopamine transportation were observed. It is known that dopaminergic neurons are more vulnerable to oxidative stress, which has been identified as potential mechanisms in the toxicity of some FR (Hendriks et al. 2014; Pellacani et al. 2014; Tagliaferri et al. 2010; Wu et al. 2016). Genes involved in oxidative stress were not altered in our transcriptomics data (data not shown), but the time points of sample collection might not be optimal to observe these effects.

Astrocytes are considered key participants in the brain maturation for providing neuronal structural, trophic and metabolic support, synthesis of growth factors and defense mechanisms, and for influencing synapse formation (Barker and Ullian 2008). Thus, the adverse effect of chemicals on astrocytes may interfere with the morphological development and functional performance of neurons in the CNS. Aiming to assess glial toxicity due to FR exposure, the gene expression of two specific markers for mature astrocytes were evaluated: glial fibrillary acidic protein (*gfap*) and the calcium–zinc-binding protein s100 beta (*s100β*). Both genes were significantly upregulated after exposure to all OPFRs. Gfap is necessary for many important processes in the CNS correlated with neuronal survival (Liedtke et al. 1996; Tardy et al. 1990). Increased gfap is an indication of gliosis/activated astrocytes, a common response of glial cells to neuronal injury and implicated in several neurological disorders such as schizophrenia, bipolar disorder and depression (Johnston-Wilson et al. 2000). In the developing CNS, s100β is believed to give support to growth, survival and differentiation of neurons (Wang and Bordey 2008), but also to play an important role in the recovery of the CSN after injury (Yardan et al. 2011). It is used as a biomarker of brain damage and as a parameter of activation of astrocytes (Esposito et al. 2006). The elevated level of s100β may also by itself produce adverse effects, including overgrowth of dystrophic neurites (Griffin et al. 1995), and is related to the occurrence of various neuropathologies (Yardan et al. 2011). The data from a study on zebrafish exposed to OPFRs are in concordance with our results by showing an increase in *gfap* expression (Sun et al. 2016).

In addition, the gene expression of the neural precursor marker *nestin* was significantly upregulated after the exposure to OPFRs. In the normal course of neurodevelopment, astrocytes and neurons differentiate from neural precursor cells leading to a decrease in expression of nestin over time (Shaltouki et al. 2013). However, upon injury to the CNS, it is transiently re-expressed in activated astrocytes (Brook et al. 1999; Chen et al. 2002; Michalczyk and Ziman 2005) and is therefore recognized as a sensitive marker for activated astrocytes (Hogberg et al. 2010, 2009). The upregulation of nestin in reactive astroglia has been observed in several diseases, e.g., cerebral ischemia, hippocampal excitotoxicity lesions, traumatic brain injury, and after MPTP (1-methyl-4-phenyl-1,2,3,6-tetrahydropyridine) exposure (Chen et al. 2002; Wei et al. 2002). It is likely that the increase in *nestin* expression observed in this study is due to reactivated astrocytes as also other glia markers were increased.

As a response to injury or chemical insult, glia cells (astrocytes and microglia) become activated and begin to produce different proinflammatory and neurotoxic substances such as cytokines and free radicals (Bal-Price and Brown 2001). TNF-α is a cytokine considered to be a primary proinflammatory mediator capable of playing a dual functional role by promoting tissue regeneration/growth and destruction (Wajant et al. 2003). IL-6 is another factor that exerts distinct functions on the CNS by participating in inflammatory responses and infections, and modulating neural processes (Abreu et al. 2018; Scheller et al. 2011). It has been shown that IL-6 influences the differentiation of neurons and astrocytes (Oh et al. 2010), but it can also be neurotoxic and cause neuronal death (Brown and Bal-Price 2003; Conroy et al. 2004). The overexpression of IL-6 has been linked to the onset of neurodevelopmental and neurodegenerative diseases and mental disorders such as schizophrenia and autism (Conroy et al. 2004; Smith et al. 2007; Wei et al. 2012). We observed a significant upregulation of the *il-6* mRNA after exposure to most OPFR at *DIV*21. After exposure to IPP, the gene expression of *tnf-α* was also increased. This indicates the onset of an inflammatory response in the 3D model after exposure to OPFR, possible as a secondary effect due to neuronal injury. Only exposure to TMPP led to possible microglia proliferation and activation as shown by upregulation in the *csf1r* and *aif1* gene expression. It should be noted that the experimental setup is not ideal to detect an inflammatory response, as cytokine release is rapid within hours after a trigger and our focus was on prolonged effects on development after long-term exposure. In addition, the microglia population, that is mainly responsible for cytokine release, is minor in the model; thus the cytokines levels can be close to the detection limits. However, transcriptomics data for IPP showed a clear increase in genes involved in inflammatory pathways and implies that the immune system plays a role in the the toxicity of OPFR. Moreover, AOP 13 (https://aopwiki.org/aops/13) links NMDA receptor inhibition during brain development to neuroinflammation and impairment of learning and memory (Villeneuve et al. 2018). Critical effects observed in this study were decreased gene expression of the NMDA receptor and glutamate levels that can be linked to the observed neuroinflammation.

This is the first time that potential underlying pathway(s) associated with developmental neurotoxicity of these OPFR have been investigated by utilizing a three-dimensional rat primary neural model. Although there are some limitations with the cell model including but not limited to kinetics, metabolism, being non-human, nonetheless it is valuable in showing how alternate streams may aid toward mechanistic understanding of classes of compounds. The advent of human brainspheres (Pamies et al. 2017) now enables similar studies in human cell models (Pamies et al. 2018; Zhong et al. 2020) work on FR is ongoing. This model also captures several end points that are not currently evaluated in traditional DNT guideline studies including, but not limited to the role of neurotransmitters and critical receptors that have previously been implicated in neurodevelopment (e.g. glutamate and GABA). Importantly, using in vitro* to *in vivo extrapolation, these findings suggest that activity is noted at relevant human exposures (within model constraints) (Blum et al. 2019). Studies are underway at the National Toxicology Program to evaluate a couple of these compounds using traditional guideline DNT studies to help provide in vivo anchors. Nonetheless, it is not feasible or practical to perform guideline studies on every member of a class of compounds. Hence, approaches such as these in combination with other assays using a DNT battery (Aschner et al. 2017; Bal-Price et al. 2018; Behl et al. 2015; Fritsche et al. 2017) complement traditional animal testing by providing guidance on prioritization, and shedding light on possible mechanistic understanding which may contribute to putative AOPs, which is currently not a part of standard guideline DNT testing.

## Electronic supplementary material

Below is the link to the electronic supplementary material.

Supplementary file1 TaqMan Primers identification. RT-qPCR means log2-fold change ± SD, from at least three independent experiments performed in 2-3 replicates after exposure to FR. Metabolomics means log2-fold change ± SD, from at least two independent experiments performed in 4-6 replicates after exposure to FR. Differences between treated and non-treated groups were assessed by two-way ANOVA (GraphPad Prism 8.4.3), followed by Bonferroni’s Comparison Post hoc test including correction for multiple testing. Post hoc test was only performed vs. controls. Statistical significance is indicated as follow *p < 0.05. Transcriptomics data after exposure to IPP  1µM at 21DIV (XLSX 779 kb)

## References

[CR1] Abreu CM, Gama L, Krasemann S (2018). Microglia increase inflammatory responses in iPSC-derived human brainspheres. Front Microbiol.

[CR2] Alakkas A, Ellis RJ, Watson CW (2019). White matter damage, neuroinflammation, and neuronal integrity in HAND. J Neurovirol.

[CR3] Alepee N, Bahinski A, Daneshian M (2014). State-of-the-art of 3D cultures (organs-on-a-chip) in safety testing and pathophysiology. Altex.

[CR4] Alzualde A, Behl M, Sipes NS (2018). Toxicity profiling of flame retardants in zebrafish embryos using a battery of assays for developmental toxicity, neurotoxicity, cardiotoxicity and hepatotoxicity toward human relevance. Neurotoxicol Teratol.

[CR5] Ariyannur PS, Moffett JR, Manickam P (2010). Methamphetamine-induced neuronal protein NAT8L is the NAA biosynthetic enzyme: implications for specialized acetyl coenzyme A metabolism in the CNS. Brain Res.

[CR6] Aschner M, Ceccatelli S, Daneshian M (2017). Reference compounds for alternative test methods to indicate developmental neurotoxicity (DNT) potential of chemicals: example lists and criteria for their selection and use. Altex.

[CR7] Bailey JM, Levin ED (2015). Neurotoxicity of FireMaster 550(R) in zebrafish (Danio rerio): chronic developmental and acute adolescent exposures. Neurotoxicol Teratol.

[CR8] Bal-Price A, Brown GC (2001). Inflammatory neurodegeneration mediated by nitric oxide from activated glia-inhibiting neuronal respiration, causing glutamate release and excitotoxicity. J Neurosci.

[CR9] Bal-Price A, Crofton KM, Leist M (2015). International STakeholder NETwork (ISTNET): creating a developmental neurotoxicity (DNT) testing road map for regulatory purposes. Arch Toxicol.

[CR10] Bal-Price A, Crofton KM, Sachana M (2015). Putative adverse outcome pathways relevant to neurotoxicity. Crit Rev Toxicol.

[CR11] Bal-Price A, Hogberg HT, Crofton KM (2018). Recommendation on test readiness criteria for new approach methods in toxicology: exemplified for developmental neurotoxicity. Altex.

[CR12] Bal-Price AK, Hogberg HT, Buzanska L, Coecke S (2010). Relevance of in vitro neurotoxicity testing for regulatory requirements: challenges to be considered. Neurotoxicol Teratol.

[CR13] Barbin G, Pollard H, Gaiarsa JL, Ben-Ari Y (1993). Involvement of GABAA receptors in the outgrowth of cultured hippocampal neurons. Neurosci Lett.

[CR14] Barker AJ, Ullian EM (2008). New roles for astrocytes in developing synaptic circuits. Commun Integr Biol.

[CR15] Baslow MH, Suckow RF, Gaynor K (2003). Brain damage results in down-regulation of N-acetylaspartate as a neuronal osmolyte. Neuromolecular Med.

[CR16] Behl M, Hsieh JH, Shafer TJ (2015). Use of alternative assays to identify and prioritize organophosphorus flame retardants for potential developmental and neurotoxicity. Neurotoxicol Teratol.

[CR17] Ben-Ari Y (2002). Excitatory actions of gaba during development: the nature of the nurture. Nat Rev Neurosci.

[CR18] Ben-Ari Y, Gaiarsa JL, Tyzio R, Khazipov R (2007). GABA: a pioneer transmitter that excites immature neurons and generates primitive oscillations. Physiol Rev.

[CR19] Bergh C, Torgrip R, Emenius G, Ostman C (2011). Organophosphate and phthalate esters in air and settled dust—a multi-location indoor study. Indoor Air.

[CR20] Birnbaum LS, Staskal DF (2004). Brominated flame retardants: cause for concern?. Environ Health Perspect.

[CR21] Bjorling-Poulsen M, Andersen HR, Grandjean P (2008). Potential developmental neurotoxicity of pesticides used in Europe. Environ Health.

[CR22] Blanke ML, VanDongen AMJ (2009) Activation mechanisms of the NMDA receptor. In: Van Dongen AM (ed) Biology of the NMDA receptor, Chap 13, CRC Press/Taylor & Francis, Boca Raton (FL). PMID: 21204408 21204408

[CR23] Blum A, Behl M, Birnbaum LS (2019). Organophosphate ester flame retardants: are they a regrettable substitution for polybrominated diphenyl ethers?. Environ Sci Technol Lett.

[CR24] Brook GA, Perez-Bouza A, Noth J, Nacimiento W (1999). Astrocytes re-express nestin in deafferented target territories of the adult rat hippocampus. NeuroReport.

[CR25] Brown GC, Bal-Price A (2003). Inflammatory neurodegeneration mediated by nitric oxide, glutamate, and mitochondria. Mol Neurobiol.

[CR26] Burke RD, Todd SW, Lumsden E (2017). Developmental neurotoxicity of the organophosphorus insecticide chlorpyrifos: from clinical findings to preclinical models and potential mechanisms. J Neurochem.

[CR27] Busse S, Brix B, Kunschmann R, Bogerts B, Stoecker W, Busse M (2014). N-methyl-d-aspartate glutamate receptor (NMDA-R) antibodies in mild cognitive impairment and dementias. Neurosci Res.

[CR28] Carlson NG, Wieggel WA, Chen J, Bacchi A, Rogers SW, Gahring LC (1999). Inflammatory cytokines IL-1 alpha, IL-1 beta, IL-6, and TNF-alpha impart neuroprotection to an excitotoxin through distinct pathways. J Immunol.

[CR29] Ceresana (2018) Flame retardants market report. https://www.ceresana.com/en/market-studies/chemicals/flame-retardants/ceresana-market-study-flame-retardants.html

[CR30] Chen LW, Wei LC, Qiu Y (2002). Significant up-regulation of nestin protein in the neostriatum of MPTP-treated mice. Are the striatal astrocytes regionally activated after systemic MPTP administration?. Brain Res.

[CR31] Chitturi J, Li Y, Santhakumar V, Kannurpatti SS (2018). Early behavioral and metabolomic change after mild to moderate traumatic brain injury in the developing brain. Neurochem Int.

[CR32] Coecke S, Goldberg AM, Allen S (2007). Workgroup report: incorporating in vitro alternative methods for developmental neurotoxicity into international hazard and risk assessment strategies. Environ Health Perspect.

[CR33] Commission E (2018) Commission Regulation (EU) laying down ecodesign requirements for electronic displays pursuant to directive 2009/125/EC of the European Parliament and of the Council, amending Commission Regulation (EC) No 1275/2008 and repealing Commission Regulation (EC) 642/2009.

[CR34] Conroy SM, Nguyen V, Quina LA (2004). Interleukin-6 produces neuronal loss in developing cerebellar granule neuron cultures. J Neuroimmunol.

[CR35] Costa LG, Giordano G (2007). Developmental neurotoxicity of polybrominated diphenyl ether (PBDE) flame retardants. Neurotoxicology.

[CR1000] Deininger MH, Meyermann R, Schluesener HJ (2002). The allograft inflammatory factor-1 family of proteins. FEBS Lett.

[CR36] Dingemans MM, Ramakers GM, Gardoni F (2007). Neonatal exposure to brominated flame retardant BDE-47 reduces long-term potentiation and postsynaptic protein levels in mouse hippocampus. Environ Health Perspect.

[CR37] Diserens G, Vermathen M, Zurich MG, Vermathen P (2018). Longitudinal investigation of the metabolome of 3D aggregating brain cell cultures at different maturation stages by (1)H HR-MAS NMR. Anal Bioanal Chem.

[CR38] Dishaw LV, Hunter DL, Padnos B, Padilla S, Stapleton HM (2014). Developmental exposure to organophosphate flame retardants elicits overt toxicity and alters behavior in early life stage zebrafish (Danio rerio). Toxicol Sci.

[CR39] Dishaw LV, Macaulay LJ, Roberts SC, Stapleton HM (2014). Exposures, mechanisms, and impacts of endocrine-active flame retardants. Curr Opin Pharmacol.

[CR40] EPA U(1998) Health effects guidelines OPPTS 870.6300, currently the EPA website is in development. https://nepis.epa.gov/Exe/ZyNET.exe/P100G6UI.TXT?ZyActionD=ZyDocument&Client=EPA&Index=1995+Thru+1999&Docs=&Query=&Time=&EndTime=&SearchMethod=1&TocRestrict=n&Toc=&TocEntry=&QField=&QFieldYear=&QFieldMonth=&QFieldDay=&IntQFieldOp=0&ExtQFieldOp=0&XmlQuery=&File=D%3A%5Czyfiles%5CIndex%20Data5C95thru99%5CTxt%5C00000033%5CP100G6UI.txt&User=ANONYMOUS&Password=anonymous&SortMethod=h%7C-&MaximumDocuments=1&FuzzyDegree=0&ImageQuality=r75g8/r75g8/x150y150g16/i425&Display=hpfr&DefSeekPage=x&SearchBack=ZyActionL&Back=ZyActionS&BackDesc=Results%20page&MaximumPages=1&ZyEntry=1&SeekPage=x&ZyPURL. Accessed Sept 24 2020

[CR41] EPA US (2005) Furniture Flame Retardancy Partnership: Environmental Profiles of Chemical Flame-Retardant Alternatives for Low-Density Polyurethane Foam, Volume1, EPA 742-R-05-002A. https://www.epa.gov/sites/production/files/2013-12/documents/ffr_foam_alternatives_vol1.pdf. Accessed Sept 24 2020

[CR42] EPA US (2008) Toxicological Review of 2,2’,4,4’-Tetrabromodiphenyl ether. US Environmental Protection Agency, EPA/635/R-07/005F. https://cfpub.epa.gov/ncea/iris/iris_documents/documents/toxreviews/1010tr.pdf. Accessed Sept 24 2020

[CR43] Eriksson P, Viberg H, Jakobsson E, Orn U, Fredriksson A (2002). A brominated flame retardant, 2,2',4,4',5-pentabromodiphenyl ether: uptake, retention, and induction of neurobehavioral alterations in mice during a critical phase of neonatal brain development. Toxicol Sci.

[CR44] Esposito G, De Filippis D, Cirillo C, Sarnelli G, Cuomo R, Iuvone T (2006). The astroglial-derived S100beta protein stimulates the expression of nitric oxide synthase in rodent macrophages through p38 MAP kinase activation. Life Sci.

[CR45] Evergren E, Benfenati F, Shupliakov O (2007). The synapsin cycle: a view from the synaptic endocytic zone. J Neurosci Res.

[CR46] Fabregat A, Jupe S, Matthews L (2018). The Reactome Pathway Knowledgebase. Nucleic Acids Res.

[CR47] Feo ML, Gross MS, McGarrigle BP (2013). Biotransformation of BDE-47 to potentially toxic metabolites is predominantly mediated by human CYP2B6. Environ Health Perspect.

[CR48] Ferreira A, Rapoport M (2002). The synapsins: beyond the regulation of neurotransmitter release. Cell Mol Life Sci.

[CR49] Flaskos J, McLean WG, Fowler MJ, Hargreaves AJ (1998). Tricresyl phosphate inhibits the formation of axon-like processes and disrupts neurofilaments in cultured mouse N2a and rat PC12 cells. Neurosci Lett.

[CR50] Forsby A, Bal-Price AK, Camins A (2009). Neuronal in vitro models for the estimation of acute systemic toxicity. Toxicol In Vitro.

[CR51] Fritsche E, Crofton KM, Hernandez AF (2017). OECD/EFSA workshop on developmental neurotoxicity (DNT): The use of non-animal test methods for regulatory purposes. Altex.

[CR52] Gaetz W, Bloy L, Wang DJ (2014). GABA estimation in the brains of children on the autism spectrum: measurement precision and regional cortical variation. Neuroimage.

[CR53] Ganguly K, Schinder AF, Wong ST, Poo M (2001). GABA itself promotes the developmental switch of neuronal GABAergic responses from excitation to inhibition. Cell.

[CR54] Gant DB, Eldefrawi ME, Eldefrawi AT (1987). Action of organophosphates on GABAA receptor and voltage-dependent chloride channels. Fundam Appl Toxicol.

[CR55] Glazer L, Hawkey AB, Wells CN (2018). Developmental exposure to low concentrations of organophosphate flame retardants causes life-long behavioral alterations in zebrafish. Toxicol Sci.

[CR56] Gogolla N, Leblanc JJ, Quast KB, Sudhof TC, Fagiolini M, Hensch TK (2009). Common circuit defect of excitatory-inhibitory balance in mouse models of autism. J Neurodev Disord.

[CR57] Goldstein ME, Sternberger NH, Sternberger LA (1987). Phosphorylation protects neurofilaments against proteolysis. J Neuroimmunol.

[CR58] Grandjean P, Landrigan PJ (2006). Developmental neurotoxicity of industrial chemicals. Lancet.

[CR59] Grandjean P, Landrigan PJ (2014). Neurobehavioural effects of developmental toxicity. Lancet Neurol.

[CR60] Griffin WS, Yeralan O, Sheng JG (1995). Overexpression of the neurotrophic cytokine S100 beta in human temporal lobe epilepsy. J Neurochem.

[CR61] Gupta RP, Lin WW, Abou-Donia MB (1999). Enhanced mRNA expression of neurofilament subunits in the brain and spinal cord of diisopropyl phosphorofluoridate-treated hens. Biochem Pharmacol.

[CR62] Hammel SC, Hoffman K, Webster TF, Anderson KA, Stapleton HM (2016). Measuring personal exposure to organophosphate flame retardants using silicone wristbands and hand wipes. Environ Sci Technol.

[CR63] Harrill JA, Robinette BL, Mundy WR (2011). Use of high content image analysis to detect chemical-induced changes in synaptogenesis in vitro. Toxicol In Vitro.

[CR64] Hausherr V, van Thriel C, Krug A, Leist M, Schobel N (2014). Impairment of glutamate signaling in mouse central nervous system neurons in vitro by tri-ortho-cresyl phosphate at noncytotoxic concentrations. Toxicol Sci.

[CR65] Haydar TF, Wang F, Schwartz ML, Rakic P (2000). Differential modulation of proliferation in the neocortical ventricular and subventricular zones. J Neurosci.

[CR66] Hendriks HS, Meijer M, Muilwijk M, van den Berg M, Westerink RH (2014). A comparison of the in vitro cyto- and neurotoxicity of brominated and halogen-free flame retardants: prioritization in search for safe(r) alternatives. Arch Toxicol.

[CR67] Hoffman K, Butt CM, Webster TF (2017). Temporal trends in exposure to organophosphate flame retardants in the United States. Environ Sci Technol Lett.

[CR68] Hoffman K, Garantziotis S, Birnbaum LS, Stapleton HM (2015). Monitoring indoor exposure to organophosphate flame retardants: hand wipes and house dust. Environ Health Perspect.

[CR69] Hogberg HT, Kinsner-Ovaskainen A, Coecke S, Hartung T, Bal-Price AK (2010). mRNA expression is a relevant tool to identify developmental neurotoxicants using an in vitro approach. Toxicol Sci.

[CR70] Hogberg HT, Kinsner-Ovaskainen A, Hartung T, Coecke S, Bal-Price AK (2009). Gene expression as a sensitive endpoint to evaluate cell differentiation and maturation of the developing central nervous system in primary cultures of rat cerebellar granule cells (CGCs) exposed to pesticides. Toxicol Appl Pharmacol.

[CR71] Honegger P, Lenoir D, Favrod P (1979). Growth and differentiation of aggregating fetal brain cells in a serum-free defined medium. Nature.

[CR72] Honegger P, Monnet-Tschudi F (2001) Aggregating neural cell cultures. In: Fedoroff S, Richardson A. (eds) Protocols for neural cell culture. Springer Protocols Handbooks. Humana Press. 10.1385/1-59259-207-4:199

[CR73] Jarema KA, Hunter DL, Shaffer RM, Behl M, Padilla S (2015). Acute and developmental behavioral effects of flame retardants and related chemicals in zebrafish. Neurotoxicol Teratol.

[CR74] Jin Z, Liu L, Bian W (2009). Different transcription factors regulate nestin gene expression during P19 cell neural differentiation and central nervous system development. J Biol Chem.

[CR75] Johnston-Wilson NL, Sims CD, Hofmann JP (2000). Disease-specific alterations in frontal cortex brain proteins in schizophrenia, bipolar disorder, and major depressive disorder. The Stanley Neuropathology Consortium. Mol Psychiatry.

[CR76] Kanehisa M, Goto S (2000). KEGG: kyoto encyclopedia of genes and genomes. Nucleic Acids Res.

[CR77] Kessner D, Chambers M, Burke R, Agus D, Mallick P (2008). ProteoWizard: open source software for rapid proteomics tools development. Bioinformatics.

[CR78] Konaka K, Ueda H, Li JY, Matsumoto M, Sakoda S, Yanagihara T (2003). N-acetylaspartate to total creatine ratio in the hippocampal CA1 sector after transient cerebral ischemia in gerbils: influence of neuronal elements, reactive gliosis, and tissue atrophy. J Cereb Blood Flow Metab.

[CR79] Li K, Xu E (2008). The role and the mechanism of gamma-aminobutyric acid during central nervous system development. Neurosci Bull.

[CR80] Liedtke W, Edelmann W, Bieri PL (1996). GFAP is necessary for the integrity of CNS white matter architecture and long-term maintenance of myelination. Neuron.

[CR81] Liu XB, Murray KD, Jones EG (2004). Switching of NMDA receptor 2A and 2B subunits at thalamic and cortical synapses during early postnatal development. J Neurosci.

[CR82] Liu Y, Dyck R, Cynader M (1994). The correlation between cortical neuron maturation and neurofilament phosphorylation: a developmental study of phosphorylated 200 kDa neurofilament protein in cat visual cortex. Brain Res Dev Brain Res.

[CR83] Lu B, Greengard P, Poo MM (1992). Exogenous synapsin I promotes functional maturation of developing neuromuscular synapses. Neuron.

[CR84] Luhmann HJ, Fukuda A, Kilb W (2015). Control of cortical neuronal migration by glutamate and GABA. Front Cell Neurosci.

[CR85] Luthi A, Schwyzer L, Mateos JM, Gahwiler BH, McKinney RA (2001). NMDA receptor activation limits the number of synaptic connections during hippocampal development. Nat Neurosci.

[CR86] Maric D, Liu QY, Maric I (2001). GABA expression dominates neuronal lineage progression in the embryonic rat neocortex and facilitates neurite outgrowth via GABA(A) autoreceptor/Cl- channels. J Neurosci.

[CR87] Michalczyk K, Ziman M (2005). Nestin structure and predicted function in cellular cytoskeletal organisation. Histol Histopathol.

[CR88] Mie A, Ruden C, Grandjean P (2018). Safety of safety evaluation of pesticides: developmental neurotoxicity of chlorpyrifos and chlorpyrifos-methyl. Environ Health.

[CR89] Miller GW, Gainetdinov RR, Levey AI, Caron MG (1999). Dopamine transporters and neuronal injury. Trends Pharmacol Sci.

[CR90] Mitrasinovic OM, Grattan A, Robinson CC (2005). Microglia overexpressing the macrophage colony-stimulating factor receptor are neuroprotective in a microglial-hippocampal organotypic coculture system. J Neurosci.

[CR91] Mostafalou S, Abdollahi M (2018). The link of organophosphorus pesticides with neurodegenerative and neurodevelopmental diseases based on evidence and mechanisms. Toxicology.

[CR92] National Academies of Sciences, Engineering, and Medicine; Division on Earth and Life Studies; Board on Environmental Studies and Toxicology; Committee to Develop a Scoping Plan to Assess the Hazards of Organohalogen Flame Retardants (2019) A class approach to hazard assessment of organohalogen flame retardants. Washington (DC): National Academies Press (US). PMID: 31436945 31436945

[CR93] Nicholson JK, Everett JR, Lindon JC (2012). Longitudinal pharmacometabonomics for predicting patient responses to therapy: drug metabolism, toxicity and efficacy. Expert Opin Drug Metab Toxicol.

[CR94] Nordengen K, Heuser C, Rinholm JE, Matalon R, Gundersen V (2015). Localisation of N-acetylaspartate in oligodendrocytes/myelin. Brain Struct Funct.

[CR95] O'Brien J, Wilson I, Orton T, Pognan F (2000). Investigation of the Alamar Blue (resazurin) fluorescent dye for the assessment of mammalian cell cytotoxicity. Eur J Biochem.

[CR96] O'Rahilly R, Muller F (2008). Significant features in the early prenatal development of the human brain. Ann Anat.

[CR97] OECD (2007) Test No. 426: Developmental Neurotoxicity Study. OECD Publishing. 10.1787/9789264067394-en. Accessed Sept 24 2020

[CR98] OECD (2011) Test No. 443: extended one-generation reproductive toxicity study. OECD Publishing, Paris, France. https://www.oecd-ilibrary.org/environment/test-no-443-extended-one-generation-reproductive-toxicity-study_9789264185371-en. Accessed Sept 24 2020

[CR99] Oh J, McCloskey MA, Blong CC, Bendickson L, Nilsen-Hamilton M, Sakaguchi DS (2010). Astrocyte-derived interleukin-6 promotes specific neuronal differentiation of neural progenitor cells from adult hippocampus. J Neurosci Res.

[CR100] Oliveri AN, Bailey JM, Levin ED (2015). Developmental exposure to organophosphate flame retardants causes behavioral effects in larval and adult zebrafish. Neurotoxicol Teratol.

[CR101] Ospina M, Jayatilaka NK, Wong LY, Restrepo P, Calafat AM (2018). Exposure to organophosphate flame retardant chemicals in the U.S. general population: data from the 2013–2014 National Health and Nutrition Examination Survey. Environ Int.

[CR102] Paivalainen S, Heape AM (2007). Myelin-associated glycoprotein and galactosylcerebroside expression in Schwann cells during myelination. Mol Cell Neurosci.

[CR103] Pamies D, Barreras P, Block K (2017). A human brain microphysiological system derived from induced pluripotent stem cells to study neurological diseases and toxicity. Altex.

[CR104] Pamies D, Block K, Lau P (2018). Rotenone exerts developmental neurotoxicity in a human brain spheroid model. Toxicol Appl Pharmacol.

[CR105] Pellacani C, Tagliaferri S, Caglieri A (2014). Synergistic interactions between PBDEs and PCBs in human neuroblastoma cells. Environ Toxicol.

[CR106] Pellerin L (2003). Lactate as a pivotal element in neuron-glia metabolic cooperation. Neurochem Int.

[CR107] Perrone Capano C, Pernas-Alonso R, di Porzio U (2001). Neurofilament homeostasis and motoneurone degeneration. BioEssays.

[CR108] Phillips AL, Hammel SC, Hoffman K (2018). Children's residential exposure to organophosphate ester flame retardants and plasticizers: Investigating exposure pathways in the TESIE study. Environ Int.

[CR109] Pluskal T, Castillo S, Villar-Briones A, Oresic M (2010). MZmine 2: modular framework for processing, visualizing, and analyzing mass spectrometry-based molecular profile data. BMC Bioinformatics.

[CR110] Quarles RH (2007). Myelin-associated glycoprotein (MAG): past, present and beyond. J Neurochem.

[CR111] Rice D, Barone S (2000). Critical periods of vulnerability for the developing nervous system: evidence from humans and animal models. Environ Health Perspect.

[CR112] Rippon G, Brock J, Brown C, Boucher J (2007). Disordered connectivity in the autistic brain: challenges for the "new psychophysiology". Int J Psychophysiol.

[CR113] Roze E, Meijer L, Bakker A, Van Braeckel KN, Sauer PJ, Bos AF (2009). Prenatal exposure to organohalogens, including brominated flame retardants, influences motor, cognitive, and behavioral performance at school age. Environ Health Perspect.

[CR114] Rubenstein JL, Merzenich MM (2003). Model of autism: increased ratio of excitation/inhibition in key neural systems. Genes Brain Behav.

[CR115] Sachana M, Rolaki A, Bal-Price A (2018). Development of the Adverse Outcome Pathway (AOP): chronic binding of antagonist to N-methyl-d-aspartate receptors (NMDARs) during brain development induces impairment of learning and memory abilities of children. Toxicol Appl Pharmacol.

[CR116] Scheller J, Chalaris A, Schmidt-Arras D, Rose-John S (2011). The pro- and anti-inflammatory properties of the cytokine interleukin-6. Biochim Biophys Acta.

[CR117] Schmittgen TD, Livak KJ (2008). Analyzing real-time PCR data by the comparative C(T) method. Nat Protoc.

[CR118] Sergent-Tanguy S, Michel DC, Neveu I, Naveilhan P (2006). Long-lasting coexpression of nestin and glial fibrillary acidic protein in primary cultures of astroglial cells with a major participation of nestin(+)/GFAP(-) cells in cell proliferation. J Neurosci Res.

[CR119] Shafer TJ, Brown JP, Lynch B, Davila-Montero S, Wallace K, Friedman KP (2019). evaluation of chemical effects on network formation in cortical neurons grown on microelectrode arrays. Toxicol Sci.

[CR120] Shaltouki A, Peng J, Liu Q, Rao MS, Zeng X (2013). Efficient generation of astrocytes from human pluripotent stem cells in defined conditions. Stem Cells.

[CR121] Shaw SD, Blum A, Weber R (2010). Halogenated flame retardants: do the fire safety benefits justify the risks?. Rev Environ Health.

[CR122] Sigel E, Steinmann ME (2012). Structure, function, and modulation of GABA(A) receptors. J Biol Chem.

[CR123] Smirnova L, Hogberg HT, Leist M, Hartung T (2014). Developmental neurotoxicity—challenges in the 21st century and in vitro opportunities. Altex.

[CR124] Smith SE, Li J, Garbett K, Mirnics K, Patterson PH (2007). Maternal immune activation alters fetal brain development through interleukin-6. J Neurosci.

[CR125] Spinu N, Bal-Price A, Cronin MTD, Enoch SJ, Madden JC, Worth AP (2019). Development and analysis of an adverse outcome pathway network for human neurotoxicity. Arch Toxicol.

[CR126] Stapleton HM, Klosterhaus S, Eagle S (2009). Detection of organophosphate flame retardants in furniture foam and U.S. house dust. Environ Sci Technol.

[CR127] Stapleton HM, Misenheimer J, Hoffman K, Webster TF (2014). Flame retardant associations between children's handwipes and house dust. Chemosphere.

[CR128] Sun L, Xu W, Peng T (2016). Developmental exposure of zebrafish larvae to organophosphate flame retardants causes neurotoxicity. Neurotoxicol Teratol.

[CR129] Sundstrom L, Morrison B, Bradley M, Pringle A (2005). Organotypic cultures as tools for functional screening in the CNS. Drug Discov Today.

[CR130] Tagliaferri S, Caglieri A, Goldoni M (2010). Low concentrations of the brominated flame retardants BDE-47 and BDE-99 induce synergistic oxidative stress-mediated neurotoxicity in human neuroblastoma cells. Toxicol In Vitro.

[CR131] Tardy M, Fages C, Le Prince G, Rolland B, Nunez J (1990). Regulation of the glial fibrillary acidic protein (GFAP) and of its encoding mRNA in the developing brain and in cultured astrocytes. Adv Exp Med Biol.

[CR132] Tonnaer EL, Peters TA, Curfs JH (2010). Neurofilament localization and phosphorylation in the developing inner ear of the rat. Hear Res.

[CR133] Trapp BD, Honegger P, Richelson E, Webster HD (1979). Morphological differentiation of mechanically dissociated fetal rat brain in aggregating cell cultures. Brain Res.

[CR134] Van Horn M, Sild M, Ruthazer E (2013). D-serine as a gliotransmitter and its roles in brain development and disease. Front Cell Neurosci.

[CR135] van Vliet E, Eixarch E, Illa M (2013). Metabolomics reveals metabolic alterations by intrauterine growth restriction in the fetal rabbit brain. PLoS ONE.

[CR136] van Vliet E, Morath S, Eskes C (2008). A novel in vitro metabolomics approach for neurotoxicity testing, proof of principle for methyl mercury chloride and caffeine. Neurotoxicology.

[CR137] van Vliet E, Stoppini L, Balestrino M (2007). Electrophysiological recording of re-aggregating brain cell cultures on multi-electrode arrays to detect acute neurotoxic effects. Neurotoxicology.

[CR138] Villeneuve DL, Landesmann B, Allavena P (2018). Representing the process of inflammation as key events in adverse outcome pathways. Toxicol Sci.

[CR139] von Ehrenstein OS, Ling C, Cui X (2019). Prenatal and infant exposure to ambient pesticides and autism spectrum disorder in children: population based case-control study. BMJ.

[CR140] Vuong AM, Yolton K, Dietrich KN, Braun JM, Lanphear BP, Chen A (2018). Exposure to polybrominated diphenyl ethers (PBDEs) and child behavior: current findings and future directions. Horm Behav.

[CR141] Wajant H, Pfizenmaier K, Scheurich P (2003). Tumor necrosis factor signaling. Cell Death Differ.

[CR142] Wang DD, Bordey A (2008). The astrocyte odyssey. Prog Neurobiol.

[CR143] Wang Y, Tang JL, Xu X (2018). NMDA receptors inhibit axonal outgrowth by inactivating Akt and activating GSK-3beta via calcineurin in cultured immature hippocampal neurons. Exp Cell Res.

[CR144] Wei H, Chadman KK, McCloskey DP (2012). Brain IL-6 elevation causes neuronal circuitry imbalances and mediates autism-like behaviors. Biochim Biophys Acta.

[CR145] Wei LC, Shi M, Chen LW, Cao R, Zhang P, Chan YS (2002). Nestin-containing cells express glial fibrillary acidic protein in the proliferative regions of central nervous system of postnatal developing and adult mice. Brain Res Dev Brain Res.

[CR146] Westerholz S, de Lima AD, Voigt T (2010). Regulation of early spontaneous network activity and GABAergic neurons development by thyroid hormone. Neuroscience.

[CR147] Wiese C, Rolletschek A, Kania G (2004). Nestin expression–a property of multi-lineage progenitor cells?. Cell Mol Life Sci.

[CR148] Wishart DS, Feunang YD, Marcu A (2018). HMDB 4.0: the human metabolome database for 2018. Nucleic Acids Res.

[CR149] Wu S, Ji G, Liu J, Zhang S, Gong Y, Shi L (2016). TBBPA induces developmental toxicity, oxidative stress, and apoptosis in embryos and zebrafish larvae (Danio rerio). Environ Toxicol.

[CR150] Yamamoto H, Kamegaya E, Hagino Y (2007). Genetic deletion of vesicular monoamine transporter-2 (VMAT2) reduces dopamine transporter activity in mesencephalic neurons in primary culture. Neurochem Int.

[CR151] Yan S, Wu H, Qin J, Zha J, Wang Z (2017). Halogen-free organophosphorus flame retardants caused oxidative stress and multixenobiotic resistance in Asian freshwater clams (Corbicula fluminea). Environ Pollut.

[CR152] Yardan T, Erenler AK, Baydin A, Aydin K, Cokluk C (2011). Usefulness of S100B protein in neurological disorders. J Pak Med Assoc.

[CR153] Ye BS, Leung AOW, Wong MH (2017). The association of environmental toxicants and autism spectrum disorders in children. Environ Pollut.

[CR154] Yi F, Rouzbeh N, Hansen KB (2020). PTC-174, a positive allosteric modulator of NMDA receptors containing GluN2C or GluN2D subunits. Neuropharmacology.

[CR155] Zhang S, Ireland D, Sipes NS, Behl M, Collins ES (2019). Screening for neurotoxic potential of 15 flame retardants using freshwater planarians. Neurotoxicol Teratol.

[CR156] Zhong X, Harris G, Smirnova L (2020). Antidepressant paroxetine exerts developmental neurotoxicity in an iPSC-derived 3D human brain model. Front Cell Neurosci.

[CR157] Zimmerman JB, Anastas PT (2015). Chemistry. Toward substitution with no regrets. Science.

